# Polyfunctional CD8^+^ CD226^+^ RUNX2^hi^ effector T cells are diminished in advanced stages of chronic lymphocytic leukemia

**DOI:** 10.1002/1878-0261.13793

**Published:** 2025-01-07

**Authors:** Maryam Rezaeifar, Shima Shahbaz, Anthea C. Peters, Spencer B. Gibson, Shokrollah Elahi

**Affiliations:** ^1^ Division of Foundational Sciences, Mike Petryk School of Dentistry University of Alberta Edmonton Canada; ^2^ Division of Medical Oncology, Department of Oncology University of Alberta Edmonton Canada; ^3^ Department of Biochemistry and Medical Genetics University of Alberta Edmonton Canada; ^4^ Li Ka Shing Institute of Virology University of Alberta Edmonton Canada; ^5^ Women and Children Health Research Institute University of Alberta Edmonton Canada; ^6^ Cancer Research Institute of Northern Alberta, Faculty of Medicine and Dentistry University of Alberta Edmonton Canada

**Keywords:** CD29^+^ T cell, co‐inhibitory receptors, IL‐6 and MIP‐1β, NK‐T cell‐like

## Abstract

CD8^+^ T cells, a subset of T cells identified by the surface glycoprotein CD8, particularly those expressing the co‐stimulatory molecule CD226, play a crucial role in the immune response to malignancies. However, their role in chronic lymphocytic leukemia (CLL), an immunosuppressive disease, has not yet been explored. We studied 64 CLL patients and 25 age‐ and sex‐matched healthy controls (HCs). We analyzed the proportion of CD226‐expressing cells among different CD8^+^ T cell subsets (including naïve, central memory, effector memory, and effectors) in CLL patients, stratified by Rai stage and immunoglobulin heavy‐chain variable region gene (IgHV) mutation status. Additionally, we compared the effector functions of CD8^+^CD226^+^ cells and their CD226^−^ counterparts. We also quantified cytokine and chemokine levels in the plasma of CLL and HCs. Furthermore, we reanalyzed the publicly available bulk RNA‐seq on CD226^+^ and CD226^−^CD8^+^ T cells. Finally, we evaluated the impact of elevated cytokines/chemokines on *CD226* expression. Our results showed that *CD226*‐expressing cells were significantly decreased within the effector memory and effector CD8^+^ T cell subsets in CLL patients with advanced Rai stages and unmutated IgHV, a marker of poor prognosis. These cells displayed robust effector functions, including cytokine production, cytolytic activity, degranulation, proliferation, and migration capacity. In contrast, CD8^+^CD226^−^ T cells displayed an exhausted phenotype with reduced Runt‐related transcription factor 2 (*RUNX2*) expression. Elevated levels of interleukin‐6 (IL‐6) and macrophage inflammatory protein‐1 beta (MIP‐1β) were inversely correlated with the frequency of CD8^+^CD226^+^ T cells and may contribute to the downregulation of CD226, possibly leading to T cell dysfunction in CLL. Our findings highlight the critical role of CD8^+^CD226^+^RUNX2^hi^ T cells in CLL and suggest that their reduction is associated with disease progression and poor clinical outcomes. This study also underscores the potential of targeting IL‐6 and MIP‐1β to preserve polyfunctional CD8^+^CD226^+^ T cells as a promising immunotherapy strategy.

## Graphical abstract

CD226^+^CD8^+^ T cells express elevated levels of RUNX2, exhibit higher proliferation capacity, cytokines and cytolytic molecules expression, and migratory capacity. In contrast, CD226^−^CD8^+^ T cells display an exhausted phenotype associated with the increased expression of co‐inhibitory receptors and impaired effector functions.

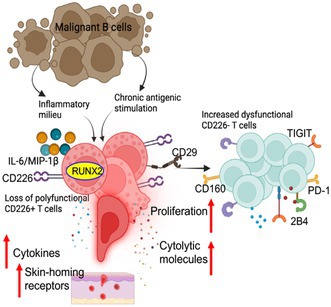

**Abbreviations**
AIRE: autoimmune regulator
AP1: activator protein 1
CBP: CREB‐binding protein
CCL: C‐C motif chemokine ligand (e.g., CCL3, CCL4L2)
CCL‐17: C‐C chemokine ligand 17
CCR: C‐C chemokine receptor (e.g., CCR4, CCR6, CCR7)
CIITA: class II transactivator
CLA: cutaneous lymphocyte antigen
CLL: chronic lymphocytic leukemia
CLL‐IPI: chronic lymphocytic leukemia international prognostic index
CM: central memory T cells
CMC1: cellular membrane component 1
CREB: cAMP response element‐binding protein
csgalnact1: chitobiosyltransferase 1
CTL: cytotoxic T lymphocytes
DKK3: dickkopf‐related protein 3
DNMT1: DNA methyltransferase 1
DUSP2: dual specificity phosphatase 2
EFF: effector T cells
EFHD2: EF‐hand domain containing 2
EM: effector memory T cells
EOMES: eomesodermin
Eotaxin: eosinophil chemotactic protein
ETV3: ETS variant 3
FC: fold change
FGF: fibroblast growth factor
FISH: fluorescence *in situ* hybridization
FOX: forkhead box (e.g., FOXP3, FOXM1, FOXO3)
Gal: galectin (e.g., Gal‐9, Gal‐3)
GM‐CSF: granulocyte‐macrophage colony‐stimulating factor
GNLY: granulysin
Gzm: granzyme (e.g., GzmB, GzmH, GzmK)
HBP1: homeobox binding protein 1
HC: healthy controls
HDAC4: histone deacetylase 4
ID1: inhibitor of DNA binding 1
IFN‐γ: interferon gamma
IgHV: immunoglobulin heavy‐chain variable region gene
IKZF1: IKAROS family zinc finger 1
IL: interleukin (e.g., IL‐1α, IL‐1β, IL‐2, IL‐4, IL‐5, IL‐6, IL‐7, IL‐8, IL‐10, IL‐12/IL‐23p40, IL‐12p70, IL‐13, IL‐15, IL‐16, IL‐17A, IL‐21, IL‐22, IL‐23, IL‐27, IL‐31)
IP‐10: interferon gamma‐induced protein 10
ITGB1: integrin beta 1
KLRG‐1: killer cell lectin‐like receptor subfamily G member 1
LGALS1: galectin‐1
Log_2_FC: Log_2_ fold change
MCP: monocyte chemoattractant protein (e.g., MCP‐1, MCP‐4)
MDC: macrophage‐derived chemokine
MHCII: major histocompatibility complex class II
MIP: macrophage inflammatory protein (e.g., MIP‐1α, MIP‐1β, MIP‐3α)
MIP‐1β: macrophage inflammatory protein‐1 beta
N: naïve T cells
NFATC2: nuclear factor of activated T cells, cytoplasmic 2
NFKB2: nuclear factor kappa B subunit 2
NKG7: natural killer group 7 protein
NK‐T cell‐like: natural killer T cell‐like
NSCLC: non‐small cell lung carcinoma
*P*adj: adjusted *P*‐value
PBMCs: peripheral blood mononuclear cells
PCA: principal component analysis
PD‐1: programmed cell death protein 1
PROK2: prokineticin 2
RB1: retinoblastoma protein
REST: repressor element‐1 silencing transcription factor
RFXANK: regulatory factor X associated protein kinase
RGS‐1: regulator of G protein signaling 1
RNA‐seq: RNA sequencing
RORγ: RAR‐related orphan receptor gamma
RUNX: runt‐related transcription factor (e.g., RUNX2, RUNX3)
SPRY‐1: sprouty protein 1
STAT: signal transducer and activator of transcription (e.g., STAT3, STAT5B)
TARC: thymus and activation‐regulated chemokine
Tcf1: T‐cell factor 1 (encoded by Tcf7)
TCR: T‐cell receptor
TFs: transcription factors
TIGIT: T cell immunoreceptor with immunoglobulin and ITIM domains
TNF: tumor necrosis factor (e.g., TNF‐α, TNF‐β)
TP53: tumor protein 53
VEGF: vascular endothelial growth factor
XCL2: X‐C motif chemokine ligand 2
Zap‐70: zeta‐chain‐associated protein kinase 70
ZNF683: zinc finger protein 683

## 1 Introduction

Chronic lymphocytic leukemia (CLL) is the most prevalent leukemia among adults in Western countries and comprises approximately 30% of leukemia cases in the USA [1]. CLL is an indolent malignancy characterized by increased production and accumulation of mature B cells co‐expressing the T‐cell antigen CD5 and B‐cell surface antigens CD19, CD20, and CD23 in the peripheral blood, bone marrow (BM), and secondary lymphoid tissues [2, 3]. The etiology of CLL is not fully understood; however, genetic factors may play an important role, as it is known to run in families [4, 5].

Generally, the prognostic features of CLL are highly variable. This poses a major barrier for clinicians in making decisions regarding the timing of therapeutic interventions in patients with CLL [6]. Several systems have been proposed by different groups for clinical staging of CLL. The Rai staging system is commonly used to determine the prognosis of CLL patients [7]. This system stratifies patients into three main groups: low‐risk (Rai stage 0), intermediate‐risk (Rai stages I/II), and high‐risk (Rai stages III/IV) [7]. Furthermore, other variables, such as the mutational status of the variable region of the immunoglobulin heavy chain (IgHV), FISH cytogenetic analysis to detect chromosomal abnormalities, B cell doubling time, and the expression of CD38, Zap‐70, and CD49d, can serve as valuable prognostic markers of CLL progression [8].

However, recent meta‐analysis data have proposed a prognostic model, the International Prognostic Index (CLL‐IPI), based on five independent prognostic factors, such as IgHV, TP53, serum 2‐microglobulin concentration, clinical stage (Rai or Binet), and age older than 65 years [9]. Based on the immune editing model, the immune system may promote tumor progression by inducing tumor variants that are adept at surviving in an immunocompetent host. This phenomenon is highly important in CLL given the indolent nature of this cancer, which allows prolonged interactions of malignant B cells with immune cells (e.g., cytotoxic CD8^+^ T cells). According to extensive studies in mice and humans, cytotoxic T lymphocytes (CTLs) play a key role in antitumor immunity [10, 11]. In line with this concept, promoting CTLs function against tumor cells is at the forefront of cancer treatment [11, 12]. However, persistent antigenic stimulation, immunoregulatory mechanisms, and soluble mediators result in the functional impairment and/or exhaustion of CTLs in cancer and chronic viral infections [11,13].

Exhausted CTLs are characterized by the loss of effector functions and changes in metabolic, epigenetic, and transcriptional profiles [11, 12]. Sustained upregulation of co‐inhibitory receptors is the hallmark of CTL exhaustion in chronic conditions, such as solid tumors [13]. Likewise, it has been reported that CTLs in CLL upregulate the expression of different co‐inhibitory receptors, including PD‐1, CD160, TIGIT, and 2B4 (CD244), resulting in their cytotoxic and proliferative impairment while retaining their ability to produce cytokines [14, 15]. Although immune checkpoint inhibitors have shown promising results for different solid tumors, their efficacy in CLL has been disappointing [14]. This has led to multiple studies exploring other strategies to reverse CD8^+^ T cell dysfunction [16], such as combination therapy with IFN‐triggering drugs and anti‐PD‐1/PD‐L1 agents or stimulation of CLL via CD40, which can reduce CD24 and CD52 expression on CD8^+^ T cells and restore their functionality [17]. Moreover, CTLs in patients with CLL exhibit impaired lytic synapse formation with malignant B cells and a defective cytotoxic function [18, 19]. Furthermore, in the presence of CLL, CTLs develop a short‐lived effector phenotype and impaired memory responses due to epigenetic reprogramming [20]. The cytotoxic function of CTLs in several hematological cancers, such as multiple myeloma and Richter syndrome, is related to disease prognosis [21, 22]. Therefore, a better understanding of how CLLs impact CTL phenotype and function is becoming increasingly significant.

While upregulation of co‐inhibitory receptors is the hallmark of CTL exhaustion, downregulation of co‐stimulatory receptors can impair CTL effector functions as well [23].

CD226 is a co‐stimulatory receptor expressed on a wide range of immune cells, but its expression is more prominent in T and NK cells [24]. CD226 competes for binding to the same receptor, CD155, as the co‐inhibitory receptors TIGIT and CD96, while CD226 delivers a stimulatory signal to immune cells [24]. TIGIT and CD96 exhibit an inhibitory and suppressive signal, ultimately resulting in CTL impairment [25]. Downregulation of CD226 on tumor‐infiltrating CTLs has been reported in several solid malignancies, such as non‐small cell lung carcinoma (NSCLC) [26] squamous cell carcinoma [25], and colorectal cancer [27]. Its downregulation has been associated with poor clinical outcomes [27] and lower response to immune checkpoint inhibitors [25, 26, 28]. Therefore, maintaining and promoting CD226 expression enhances CD8^+^ T cell function and the efficacy of immunotherapy [28]. Likewise, the loss of CD8^+^CD226^+^ T cells in multiple myeloma has been shown to be associated with suboptimal response to cyclophosphamide (CTX) and bortezomib [28, 29]. The expression of TIGIT and CD226 is not limited to CD8^+^ T cells; these markers are also present on other cell types, including malignant CLL cells. Their expression on malignant B cells has been shown to significantly impact both the function of immune cells and the progression of the disease [30]. However, to the best of our knowledge, the expression and characteristics of CD8^+^CD226^+^ T cells and their subsets have not been evaluated in CLL patients. In this study, we hypothesized that a reduction in the frequency of CD8^+^CD226^+^ T cells is associated with a poor prognosis. Therefore, we investigated the frequency and functionality of CD226^+^ compared to their negative counterparts in a cohort of CLL patients and healthy controls (HCs). Specifically, we compared the upregulated and downregulated genes in CD226^+^ versus CD226^−^CD8^+^ T cells using publicly available bulk RNA sequencing data conducted on CD226^+^ and CD226^−^ from HCs and quantified plasma‐soluble analytes in both CLL patients and HCs.

## 2 Materials and methods

### 2.1 Human study cohort

We enrolled 64 human patients with CLL from the Cross Cancer Institute at the University of Alberta and 25 age‐ and sex‐matched healthy controls (HCs). Our patients were either treatment‐naive or had not received any treatment in the past 6 months. Any treatments listed in Table S1 occurred more than 6 months prior to blood collection. Peripheral blood samples were collected from the EDTA‐containing tubes. Additionally, clinical data were collected for further analysis, including IgHV mutation status, FISH analysis, and clinical assessment using the Rai staging system (Table S1).

### 2.2 Ethics statement and consent to participate

This study was reviewed and approved by the Health Research Ethics Board of the Alberta Cancer Committee (HREBA, #CC‐17‐0307). In addition, the recruitment of HCs was approved by the Health Research Ethics Board at the University of Alberta (protocol #Pro00063463). All study subjects provided written informed consent to participate in the study, and samples were collected from October 2022 to May 2024. The study methodologies conformed to the standards set by the Declaration of Helsinki.

### 2.3 Cell isolation and purification

Peripheral blood mononuclear cells (PBMCs) from patients with CLL or HCs were isolated using Ficoll‐Paque gradients (GE Healthcare, Chicago, IL, USA) and cultured in RPMI 1640 (Sigma‐Aldrich) supplemented with 10% FBS (Sigma‐Aldrich) and 1% penicillin/streptomycin (Sigma‐Aldrich, Burlington, MA, USA), according to our routine protocols [31, 32]. Plasma was collected and stored at −80 °C for subsequent analysis.

### 2.4 Flow cytometry

Fluorochrome‐conjugated antibodies specific to human cell antigens and cytokines were purchased from BD Biosciences (Ashland, OR, USA), Thermo Fisher Scientific (Waltham, MA, USA), BioLegend (San Diego, CA, USA), or Abcam (Waltham, MA, USA). The following antibodies were used in this study: anti‐CD3 (SK7), anti‐CD4 (RPA‐T4), anti‐CD8 (RPA‐T8), anti‐CD226 (DX11), CD160 (BY55), anti‐2B4 (2–69), anti‐TIGIT (MBSA43), anti‐PD1 (EH12.1), anti‐KLRG1 (2F1/KLRG1), anti‐CD107a (H4A3), anti‐TNF‐α (MAB11), and anti‐IFN‐γ (45. B3), anti‐perforin (dG9), anti‐granzyme B (GB11), anti‐granzyme K (G3H69), anti‐CLA (HECA452; Miltenyi, Gaithersburg, MD, USA ), anti‐CCR4 (1G1), anti‐CCR6 (11A9), anti‐integrin‐7 (FIB504), anti‐CCR7 (3D12), anti‐CD45RA (HI100), anti‐granulysin (RB1), anti‐CD56^−^ (NCAM‐1), anti‐CD29 (HMb1‐1), anti‐EOMES (WD1928), anti‐FOXP3^−^ (236A/E7), anti‐T‐bet (O4‐46), anti‐RORγt (Q21–559), and anti‐RUNX2 (EPR14334; Abcam).

### 2.5 Cell culture, cytokine, and proliferation assays

Cultured PBMCs were stimulated with anti‐CD3 (UCHT1, 3 μg·mL^−1^) and anti‐CD28 (CD28.2, 1 μg·mL^−1^) antibodies in RPMI‐1640 medium in the presence of the protein transport inhibitor, Brefeldin A (BD Biosciences), for 5 h. Intracellular cytokine staining was performed as previously described [33, 34]. In some experiments, PBMCs were also treated with recombinant cytokines, such as IL‐6 (0.02 ng·mL^−1^), MIP‐1β (1 ng·mL^−1^), and IL‐2 (0.4 ng·mL^−1^), after initial stimulation with anti‐CD3/CD28 antibodies or without stimulation. For the proliferation assay, isolated total T cells were labeled with CFSE dye (Life Technologies, Carlsbad, CA, USA) and then stimulated with anti‐CD3/CD28 antibodies for 5 days before analysis, according to our protocols [35]. Unstimulated cells served as controls.

### 2.6 Mesoplex and ELISA assays

A wide range of soluble analytes was measured using the V‐Plex proinflammatory panel 1, cytokine panel 1, chemokine panel 1, and Th17 panel 1 (Meso Scale Discovery, Rockville, MD, USA), according to the manufacturer's instructions, as previously reported [36, 37]. Plasma samples were centrifuged at 2000 ***g*** for 10 min and appropriately diluted according to the specific guidelines provided for each assay. The following analytes were quantified: Eotaxin, Eotaxin‐3, FGF (basic), GM‐CSF, IFN‐γ, IL‐1α, IL‐1β, IL‐2, IL‐4, IL‐5, IL‐6, IL‐7, IL‐8, IL‐10, IL‐12/IL‐23p40, IL‐12p70, IL‐13, IL‐15, IL‐16, IL‐17A, IL‐21, IL‐22, IL‐23, IL‐27, IL‐31, IP‐10, TARC, VEGF, MCP‐1, MCP‐4, MDC, MIP‐1α, MIP‐1β, MIP‐3α, TNF‐α, TNF‐β. In addition, Galectin‐9 (Gal‐9) and Galectin‐3 (Gal‐3) were detected using the DuoSet ELISA kit (R&D, Minneapolis, MN, USA), and soluble levels of CD226 were detected using the CD226/DNAM‐1 Human ELISA Kit Sandwich ELISA Kit, according to the manufacturer's protocol.

### 2.7 Migration assay

The migration assay was performed using the CytoSelect migration assay kit (Cell Biolabs, San Diego, CA, USA), as we have reported elsewhere [32, 38]. PBMCs were starved overnight in FBS‐free culture media. The next day, FBS (10%) and recombinant human CCL‐17 (100 ng·mL^−1^) were used as chemoattractants for the CCR4 receptor [39]. Cell suspension of starved cells (0.5 × 106 cells/well) was added to the upper chamber and incubated in the incubator (37 °C, 5% CO25) for 24 h. Migrated cells in the lower chamber were harvested and quantified by flow cytometry according to the manufacturer's instructions. The migration ratio was calculated compared to the wells lacking the chemoattractant.

### 2.8 RNA‐Seq analysis

We used a publicly available RNA‐Seq database (GSE133397 and GSE135291) [28]. Kallisto with 100 permutations and bias correction was used for fragment alignment in the human cDNA database (GRCh38). Differential expression (DE) analysis was conducted by analyzing the count data using the deseq2 R package (R version 4.2.0) and transcriptional factors (TFs) using decoupler. Differentially expressed genes demonstrated corrected *P*‐value (*P*adj < 0.05) and −0.5 < log_2_‐fold change (FC) > +0.5. Principal component analysis (PCA), heat maps, volcanoes, and upset plots were generated using R scripts. Bubble plots were generated using a Python script.

### 2.9 Statistical analysis

graphpad prism (Boston, MA, USA) software was used for statistical analysis. For comparison, the non‐parametric Mann–Whitney *U* test or Wilcoxon signed‐rank test was used for non‐paired and paired datasets, respectively. The Kruskal–Wallis test, followed by Dunn's *post‐hoc* test, was used when more than two groups were compared.

## 3 Results

### 3.1 A significant reduction in the frequency of CD226^+^ effector and effector memory CD8^+^ T cells in high‐risk CLL patients

**Fig. 1:**
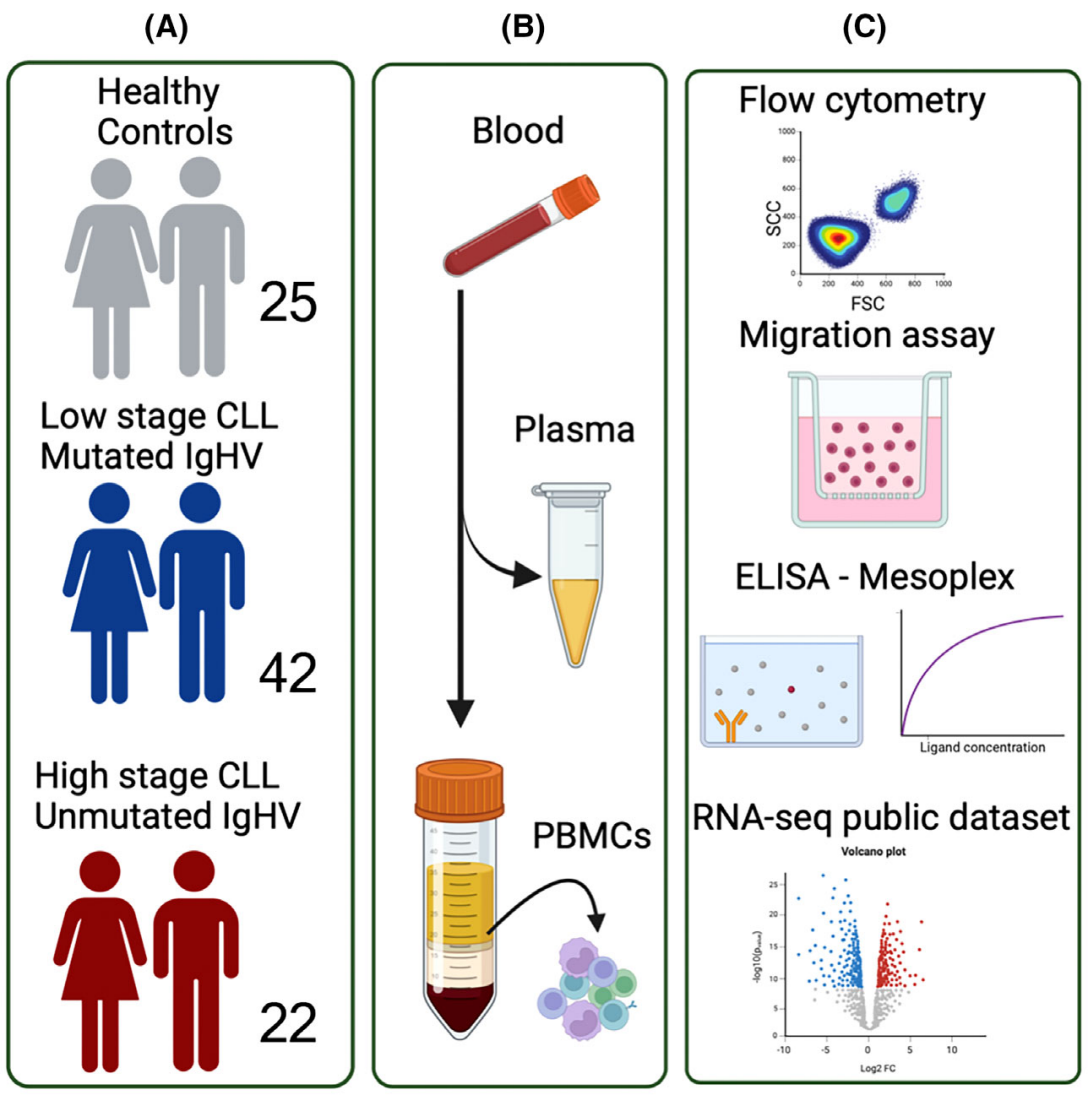
The experimental workflow. (A) Illustrates the number of study subjects, including healthy controls (25), low disease stage chronic lymphocytic leukemia (CLL) (42), and high disease stage CLL, those with unmutated variable region of the immunoglobulin heavy chain (IgHV) (22). (B) The collection of plasma and isolation of peripheral blood mononuclear cells (PBMCs) from the blood. (C) Illustrating major techniques used in the study, such as flow cytometry, migration assay, multiplex (Mesoples) and ELISA assays, and bulk RNA‐sequencing (RNA‐seq).

**Fig. 2:**
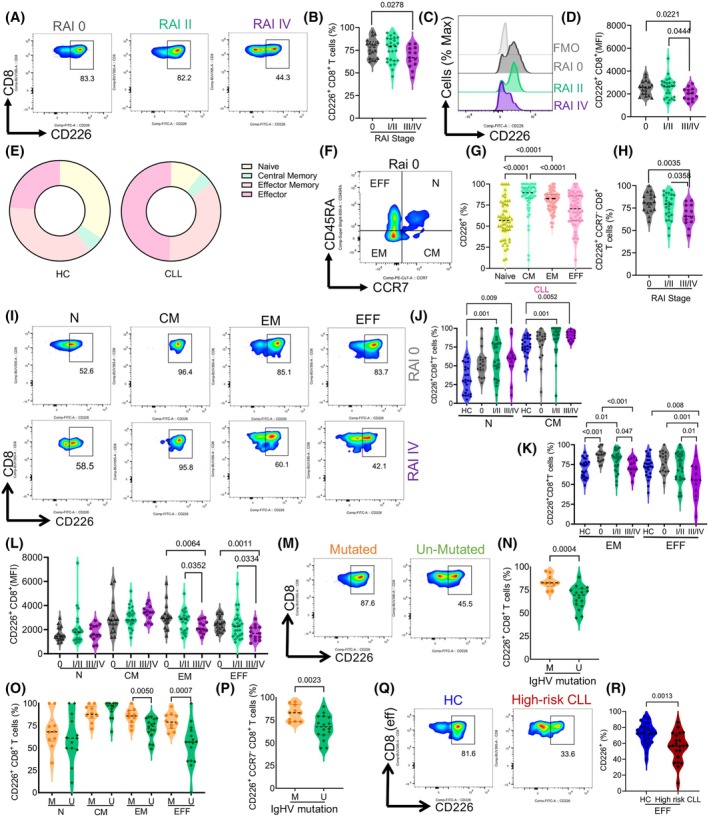
Reduction of CD8^+^CD226^+^ effector and effector memory T cells in high‐risk CLL. (A) Representative flow cytometry plots and (B) cumulative data comparing the frequency of CD8^+^CD226^+^ T cells in peripheral blood mononuclear cells (PBMCs) from chronic lymphocytic leukemia (CLL) patients in low (0), intermediate (I/II), and high (III/IV) Rai stages. (C) Representative histogram plots, and (D) cumulative data comparing the Mean Fluorescence Intensity (MFI) of CD226 in CD8^+^ T cells from CLL patients in low (0), intermediate (I/II), and high (III/IV) Rai stages. (E) Pie charts representing the median frequency of different subsets of CD8^+^ T cells in healthy controls (HC) and CLL. (F) Representative plot of different CD8^+^ T cell subsets [naïve (N; CD45RA^+^CCR7^+^), central memory (CM; CD45RA‐CCR7^+^), effector memory (EM; CD45RA‐CCR7^−^), and effector cells (EFF; CD45RA^+^CCR7^−^)]. (G) Cumulative data comparing the frequency of CD226 expression in different subsets of CD8^+^ T cells in CLL. (H) Cumulative data of CD226 frequency in CD8^+^CCR7^−^ T cells (effector and effector memory) in CLL patients in low (0), intermediate (I/II), and high (III/IV) Rai stages. (I) Representative flow cytometry plots, and (J) cumulative data of the frequency of CD226^+^ cells among N and CM CD8^+^ T cell subsets from HCs and CLL patients in low (0) and high (IV) Rai stages. (K) Cumulative data of the frequency of CD226^+^ cells among EM and EFF CD8^+^ T cell subsets from HCs and CLL patients in low (0) and high (IV) Rai stages. (L) MFI for CD226 expression in N, CM, EM, and EFF CD8^+^ T cells in CLL patients in low (0), intermediate (I/II), and high (III/IV) Rai stages. (M) Representative flow cytometry plots, and (N) cumulative data comparing the frequency of CD8^+^CD226^+^ T cells in CLL patients with mutated and unmutated immunoglobulin heavy chain (IgHV) status. (O) Cumulative data of the frequency of CD226^+^ cells within different CD8^+^ T cell subsets in CLL patients with mutated and unmutated IgHV mutational status. (P) Cumulative data of the frequency of CD226^+^ cells within CD8^+^CCR7^−^ T Cells (EM and EFF) in CLL patients with mutated (M) and unmutated (U) IgHV mutational status. (Q) Representative flow cytometry plots and (R) cumulative data comparing the frequency of CD8^+^CD226^+^ EFF T cells in PBMCs from HCs with high‐risk CLL patients (unmutated IgHV and (III/IV) Rai stages). Fluorescence minus one (FMO). The cumulative data are shown as the medians and interquartile ranges. The *P* values were measured using the Mann Whitney *U* test (N, P, R) or Kruskal–Wallis test followed by Dunn's *post‐hoc* test (B, D, G, H, J, K, L, O). Data are presented as median and interquartile ranges (IQR).

Our studies followed the procedures outlined in the experimental workflow (Fig. 1). Given the antitumor properties of CD8^+^CD226^+^ cells [27], we quantified their frequency with respect to disease stage. Interestingly, we found that the frequency of CD8^+^CD226^+^ T cells was significantly reduced in Rai stage III/IV compared to Rai stage 0 (Fig. 2A,B, and Fig. S1A) and similarly the intensity of CD226 expression (Fig. 2C,D). CD8^+^ T cells are highly heterogeneous, and each subset plays a unique role in tumor immunity [40]; therefore, we assessed the frequency of CD226^+^ expressing cells among different CD8^+^ T cell subsets with respect to the disease stage. Considering the variability of T cell subpopulations in CLL versus HCs (Fig. 2E), we measured the frequency of CD226^+^ cells among different T cell subsets, including naïve (CD45RA^+^CCR7^+^), central memory (CM; CD45RA‐CCR7^+^), effector memory (EM; CD45RA^−^CCR7^−^), and effector cells (EFF; CD45RA^+^CCR7^−^) (Fig. 2F). Interestingly, we noticed that the naïve subset had the lowest proportion of CD226^+^ cells compared to the other subsets in both CLL patients (Fig. 2G) and HCs (Fig. S1B).

Given that CD8^+^ T cells in CLL are enriched with EFF and EM subsets (Fig. 2E), we further analyzed the frequency of CD226‐expressing cells within these subsets (CD8^+^CCR7^−^). We observed a significant reduction in the frequency of CD226^+^ EFF and CD226^+^ EM CD8^+^ T cells in Rai stage III/IV CLL patients compared to those in Rai stages I/II and 0 (Fig. 2H). Given the elevated numbers of T cells in the blood circulation of CLL patients compared to HCs [14], we decided to compare CD226^+^CD8^+^ T cells between CLL and HCs. Notably, there was no significant difference in the frequency of CD226^+^CD8^+^ T cells between naïve and CM T cells in HCs and stage 0 CLL patients. However, these cells expand among naïve and CM subsets in later disease stages compared to HCs. The pattern is different in EM and EFF subsets; a higher frequency of CD226^+^ subset in EM in stages 0 and I/II might suggest a protective role for these cells. Nevertheless, as the disease progresses, the frequency of CD226^+^ cells among EM T cells reduces in CLL patients, and a significant reduction in the EFF subset was observed compared to HCs and earlier stages of the disease (Fig. 2I–K). Similarly, we found that EM and EFF T cells in the later Rai stage displayed a significantly lower intensity of CD226 expression, but this was not the case for naïve and CM CD8^+^ T cells (Fig. 2L). Considering the prognostic significance of IgHV mutation status in our study, we compared the frequency of CD226‐expressing CD8^+^ T cells in IgHV‐mutated versus unmutated patients. Although we were unable to obtain the mutation status for all patients, this was performed on only 42.2% of patients with a determined IgHV mutation. We found a notable decrease in the frequency of CD226 expressing CD8^+^ T cells in unmutated patients compared to total CD8^+^ T cells (Fig. 2M,N). We further analyzed the frequency of CD226 in different subsets of CD8^+^ T cells in patients with unmutated and mutated CLL. These analyses revealed a significant reduction in the frequency of CD226^+^ in EFF and EM CD8^+^ T cells in unmutated compared with mutated patients; however, this was not the case for CM and naive CD8^+^ T cells (Fig. 2O,P). A similar observation was made for the intensity of CD226 expression in EM and EFF CD8^+^ T cells (Fig. S1C). In light of the prominent reduction in the frequency of CD226 in both advanced‐stage and unmutated CLL patients within the EFF CD8^+^ T cell subpopulation, we compared their frequency in high‐risk CLL patients (Unmutated or Rai III/IV) to HCs. Our findings revealed a substantial reduction in CD226‐expressing EFF subsets in high‐risk CLL patients compared to HC (Fig. 2Q,R). Overall, we observe a reduction in the frequency and intensity of CD226, in particular, in the EFF subset in the advanced stages of CLL.

### 3.2 CD8^+^CD226^+^ effector T cells exhibit higher cytokine, cytolytic molecules expression, and proliferation capacity

**Fig. 3:**
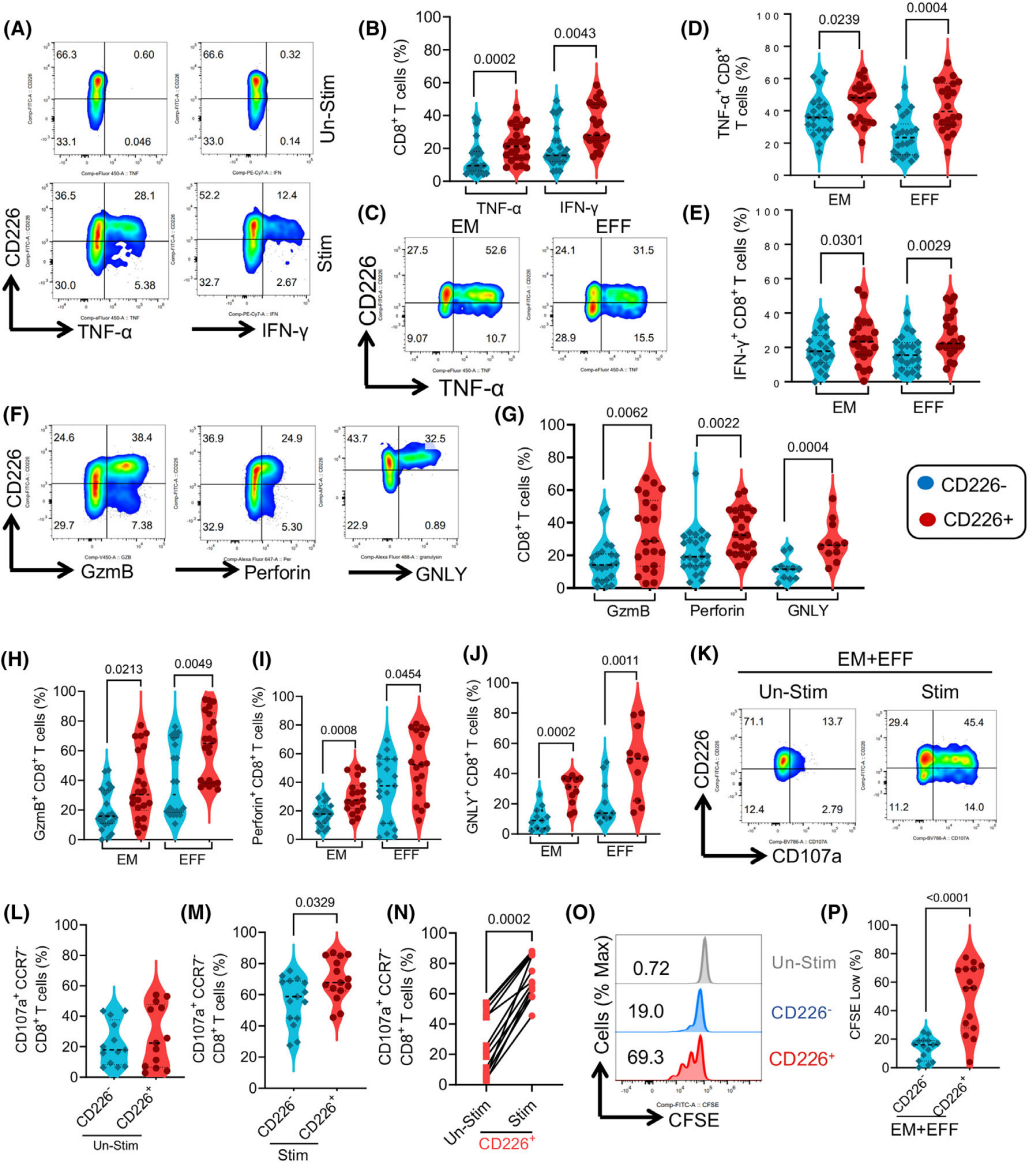
CD8^+^CD226^+^ effector T cells are highly polyfunctional. (A) Representative flow plots and (B) cumulative data of TNF‐α and IFN‐γ expression in CD226^+^ versus CD226^−^CD8^+^ T cells upon stimulation of peripheral blood mononuclear cells (PBMCs) from chronic lymphocytic leukemia (CLL) patients with anti‐CD3/CD28 antibodies. (C) Representative flow plots of TNF‐α expression in CD226^+^ versus CD226^−^ in effector memory (EM) and effector (EFF) CD8^+^ T cells from CLL patients upon stimulation. (D) Cumulative data of TNF‐α, and (E) IFN‐γ expression in CD226^+^ versus CD226^−^ in EM and EFF subsets of CD8^+^ T cells from CLL patients. (F) Representative flow plots and (G) cumulative data of perforin, Granzyme B (GzmB), and granulysin (GNLY) expression in CD226^+^ versus CD226^−^CD8 T cells from CLL patients. (H) Cumulative data of GzmB, (I) Perforin, and (J) GNLY expression in CD226^+^ versus CD226^−^ in EM and EFF subsets of CD8^+^ T cells from CLL patients. (K) Representative flow plots, and (L–N) cumulative data of the frequency of CD107a^+^ cells among CD226^+^ versus CD226^−^CD8^+^CCR7^−^ T cells from CLL patients either unstimulated (unstim) or stimulated (stim) with anti‐CD3/CD28 in the presence of protein transport inhibitor. (O) Representative flow plots and (P) cumulative data of the frequency of CFSE^low^ (proliferated) cells among CD226^+^ versus CD226^−^CD8^+^CCR7^−^ T Cells, Unstimulated (gray color) after 5‐day culture. The cumulative data are shown as the medians and interquartile ranges. The *P* values were measured using the Mann Whitney *U* test (L, M, P), the Kruskal–Wallis test followed by Dunn's *post‐hoc* test (B, D, E, G–J), or Wilcoxon test (N). Data are presented as median and interquartile ranges (IQR).

Peripheral blood mononuclear cells from CLL patients were stimulated with anti‐CD3/CD28 and subjected to intracellular cytokine staining (ICS) according to our protocols [34, 41]. Our results revealed that CD8^+^CD226^+^ T cells exhibited significantly higher IFN‐γ and TNF‐α expression than their CD226^−^ counterparts (Fig. 3A,B). Given the pronounced difference in CD226 frequency/expression between high‐ and low‐risk CLL patients within the EFF and EM CD8^+^ T cell subsets, we further investigated the consistency of this observation in the EFF and EM CD8^+^ T cell subpopulations. After examining the production of TNF‐α and IFN‐γ by various CD8^+^ T cell subsets, we noted that CD226‐expressing cells consistently exhibited significantly greater levels of TNF‐α and IFN‐γ than their negative counterparts in both EFF and EM subsets (Fig. 3C–E).

Additionally, we found that the presence of CD226 was associated with increased expression of cytolytic molecules, including perforin, GzmB, and granulysin (GNLY) expression (Fig. 3F,G). A similar pattern was observed for EFF and EM subsets for the expression of these cytolytic molecules compared to their CD226^−^ counterparts in CLL patients (Fig. 3H–J).

When the degranulation capacity (CD107a) was evaluated, we found no significant difference in CD107a expression between CD226^+^ and CD226^−^CD8^+^CCR7^−^ T cells in the absence of TCR stimulation (Fig. 3K,L). However, upon TCR stimulation (anti‐CD3/CD28 antibodies), CD226^+^ EFF and EM T cells exhibited enhanced degranulation capacity compared with CD226^−^ EFF and EM T cells (Fig. 3K,M,N).

Additionally, we found that CD226^+^ EFF and EM T cells displayed a significantly greater proliferative capacity than their CD226^−^ counterparts in CLL patients (Fig. 3O,P). Taken together, our findings support the polyfunctionality of CD8^+^CD226^+^ T cells.

### 3.3 CD8^+^CD226^+^ T cells display a CD8 NK‐T cell‐like feature

**Fig. 4:**
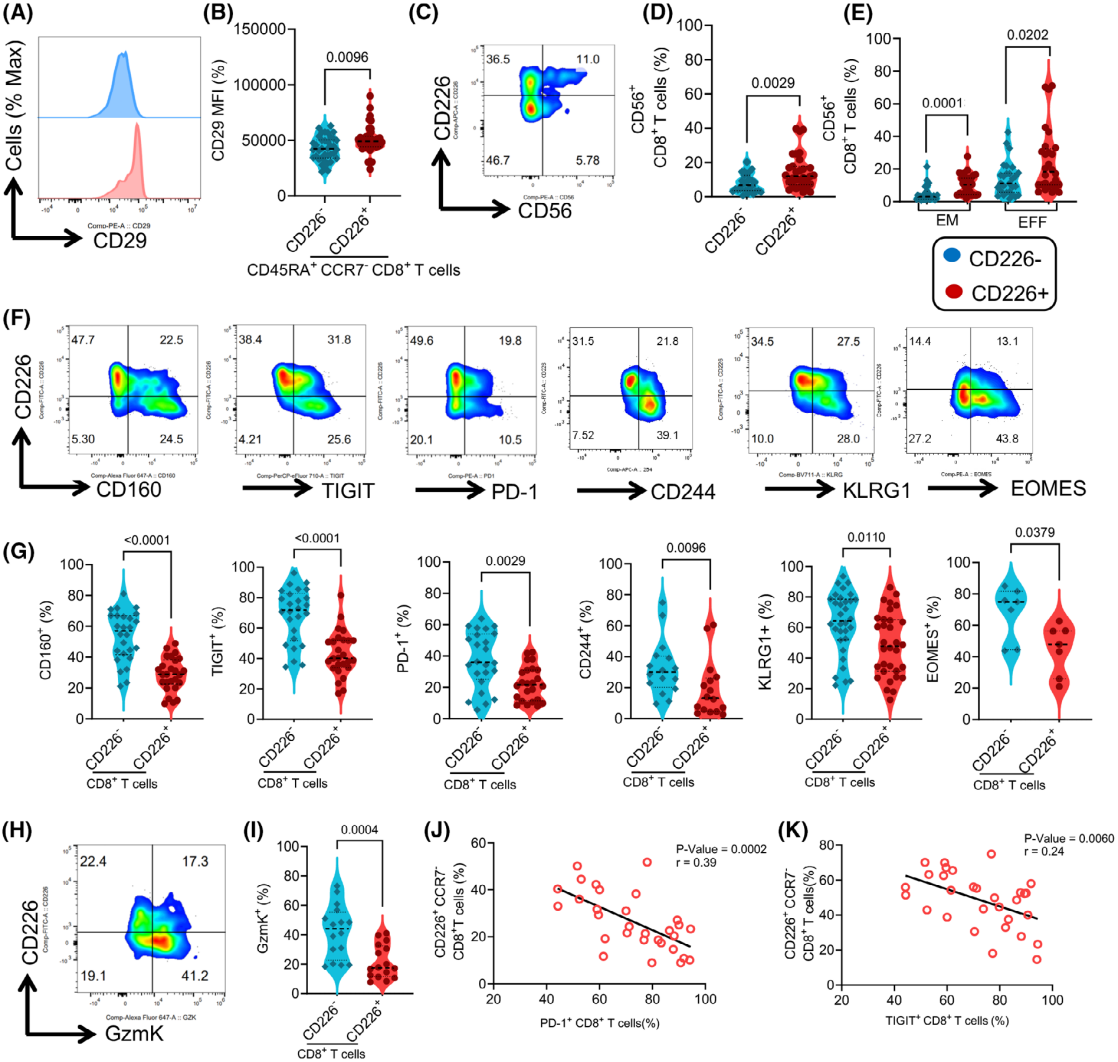
CD8^+^CD226^+^ effector T cells express CD29 but CD226^−^ exhibit features of exhausted T cells. (A) Representative histogram plots and (B) cumulative data of the intensity of CD29 in CD226^+^ versus CD226^−^ effector CD8^+^ T cells in chronic lymphocytic leukemia (CLL) patients. (C) Representative flow plot, and (D) cumulative data comparing the frequency of CD56 in CD226^+^ versus CD226^−^CD8^+^ T cell. (E) Cumulative data of the frequency of CD56 in CD226^+^/CD226^−^ effector memory (EM) and effector (EFF) subsets in CLL. (F) Representative flow cytometry plots and (G) cumulative data of the frequency of CD160, TIGIT, PD‐1, CD244, KLRG1, and EOMES‐expressing cells among CD226^+^ versus CD226^−^CD8^+^ T cell. (H) Representative flow plot and (I) cumulative data comparing the frequency of Granzyme K (GzmK) in CD226^+^ versus CD226^−^CD8^+^ T cell. (J) The correlation between percentages of CD226^+^CD8^+^ T cells with PD‐1^+^CD8^+^ T cells and (K) TIGIT^+^CD8^+^ T cells in CLL patients. The cumulative data are shown as the medians and interquartile ranges. The *P* values were measured using the Mann Whitney *U* test (B, D, G, I), the Kruskal–Wallis test followed by Dunn's *post‐hoc* test (E), and linear regression (J, K). Data are presented as median and interquartile ranges (IQR).

CD8^+^CD29^+^ T cells have been reported as IFN‐γ‐producing T cells [35], and given the similar phenotype of CD8^+^CD226^+^ T cells in our cohort, we assessed the expression of CD29 in CD8^+^ T cells. However, we did not find any significant difference in the frequency and intensity of CD29‐expressing cells among total CD226^+^/CD226^−^ T cells (Fig. S1D–F). However, we observed a significant upregulation in CD29 expression among CD8^+^CD226^+^ T EFF cells compared to their negative counterparts (Fig. 4A,B and Fig. S1G). Although the majority of CD8^+^ EFF T cells in patients with CLL expressed CD29, CD8^+^CD226^+^ EFF T cells expressed a significantly heightened intensity of CD29, categorizing them as CD29^hi^CD8^+^CD226^+^ EFF T cells (Fig. 4A,B). Given the pronounced cytotoxicity, we investigated whether CD8^+^CD226^+^ T cells were enriched in NK‐T‐like cells marked by CD56 [42, 43]. Further analysis revealed a significantly higher proportion of CD56‐expressing cells among CD8^+^CD226^+^ T cells compared to their CD226^−^ counterparts (Fig. 4C–E). Notably, despite the significant proportion of CD56‐expressing cells among the EFF subset, CD226^+^ EFF T cells maintained a significantly higher frequency of CD56 than their negative counterparts (Fig. 4E). Our findings support the presence of an NK‐T‐like cell phenotype within the CD8^+^CD226^+^ T cell subset.

### 3.4 CD226^−^ but not CD8^+^CD226^+^ effector T cells exhibit features of exhausted T cells

As the sustained upregulation of co‐inhibitory receptors is associated with CD8^+^ T cell exhaustion [44], we evaluated their expression in CD226^+^ and CD226^−^CD8^+^ T cells. Given our previous work and that of others, which have highlighted CD160, TIGIT, PD‐1, and CD244 as the most prominent co‐inhibitory receptors expressed among CD8^+^ T cells in CLL patients [14, 45], we focused on these co‐inhibitory receptors. We found significant enrichment of CD160, 2B4, TIGIT, PD‐1, and KLRG‐1‐expressing T cells among total CD226^−^CD8^+^ T cells compared to their CD226^+^ counterparts (Fig. 4F,G), which was also the case among the EFF and EM subsets, except for PD‐1 (Fig. S1H).

CD8^+^ T cell exhaustion is also accompanied by altered expression of transcriptional factors [46], among which EOMES and T‐bet play pivotal roles. T‐bet and EOMES imbalance can result in immune system dysfunction, with high EOMES expression in CD8^+^ T cells being associated with exhaustion. However, EOMES plays an essential role in the maintenance of memory CD8^+^ T cells [47]. We evaluated the expression of these transcription factors in CD8^+^ T cells. Consistent with previous findings, we observed significantly higher percentages of CD226^−^CD8^+^ T cells expressing EOMES than CD8^+^CD226^+^ T cells (Fig. 4F,G). However, this was not the case for T‐bet expression (Fig. S1I). We also examined the expression of transcriptional factors for regulatory T cells (FOXP3) and Th17 cells (RORγt) in CD226^+^ and CD226^−^CD8^+^ T cells, but no differences were noted (Fig. S1J,K).

It is worth mentioning that a recent study has reported the presence of highly exhausted GZMK^+^CD8^+^ T cells in the bone marrow of relapsed/refractory multiple myeloma patients [48]. In addition, GZMK^+^CD8^+^ exhausted T cells are linked to a poor prognosis in multiple myeloma and inflammaging [22, 49]. Therefore, we compared the expression of GZMK in CD226^+^ and their CD226^−^ counterparts and found significantly higher GZMK expression in total CD226^−^CD8^+^ T cells (Fig. 4H,I) and their EFF and EM subsets (Fig. S1H).

Given the significant reduction in CD226^+^ EFF and EM T cells in high‐risk CLL patients, we further investigated the correlation between these cells and co‐inhibitory receptors. Interestingly, we found a moderate inverse correlation between the frequency of CD26^+^ EFF and EM T cells and the proportion of CD8^+^ TIGIT^+^ and CD8^+^ PD‐1^+^ T cells (Fig. 4J,K). However, this was not the case for the frequency of CD226^+^ EFF and EM T cells and CD8^+^CD160^+^ T cells in our high‐risk patients (Fig. S1L). Taken together, our results demonstrate that CD226^−^ but not CD8^+^CD226^+^ T cells display an exhausted phenotype in CLL patients.

### 3.5 CD8^+^CD226^+^ T cells display higher skin homing receptors

**Fig. 5:**
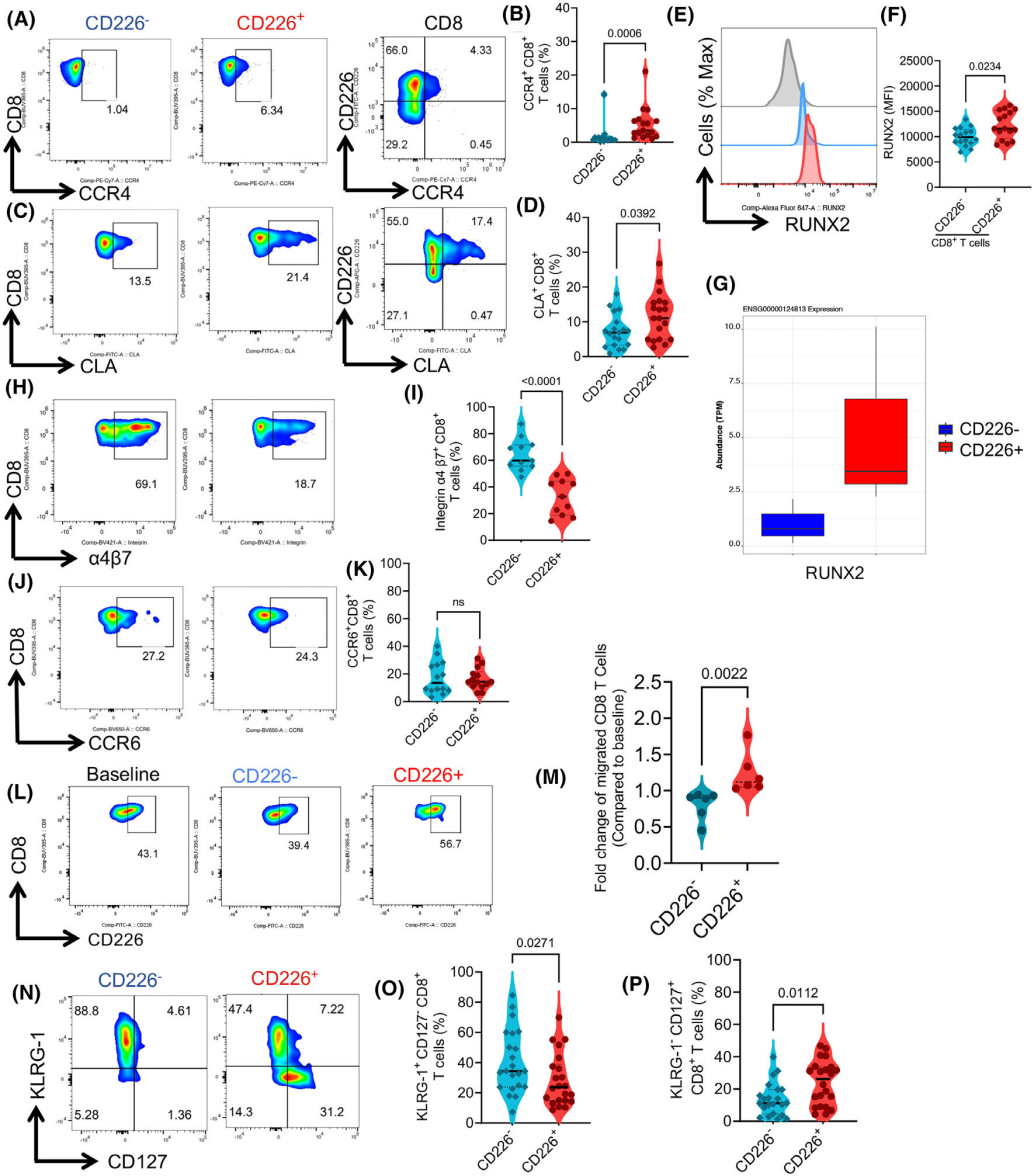
CD8^+^CD226^+^ T cells display higher skin‐homing receptors. (A) Representative flow plots of the frequency of CCR4‐expression among CD226^+^, CD226^−^, and total CD8^+^ T cells, and (B) cumulative data of the frequency of CCR4 expressing cells among CD226^+^ versus CD226^−^CD8^+^ T cells. (C) Representative flow plots of the frequency of CLA‐expression among CD226^+^, CD226^−^, and total CD8^+^ T cells, and (D) cumulative data of the frequency of CLA‐expressing cells among CD226^+^ versus CD226^−^CD8^+^ T cells. (E) Representative histogram plots and (F) cumulative data of the Mean Fluorescence Intensity (MFI) of RUNX2 expression among CD226^+^ versus CD226^−^CD8^+^ T cells. (G) Box plot showing the abundance of transcript per million (TPM) of the RUNX2 in effector CD226^−^ and CD8^+^CD226^+^ T cells. (H) Representative flow plots, and (I) cumulative data of the frequency of Integrin α4β7 expression among CD226^+^ versus CD226^−^CD8^+^ T cells in chronic lymphocytic leukemia (CLL). (J) Representative flow plots and (K) cumulative data of the frequency of CCR6‐expressing cells among CD226^+^ versus CD226^−^CD8^+^ T cells. (L) Representative flow cytometry plots and (M) cumulative data of the migratory capacity of CD226^−^ versus CD226^+^CD8^+^ T cells in response to CCL‐17. (N) Representative flow plots, and (O) cumulative data of the frequency of CD127^−^/KLRG1^+^, and (P) CD127^+^/KLRG1‐expressing cells among CD226^+^ and CD226^−^CD8^+^ T cells in CLL patients. The cumulative data are shown as the medians and interquartile ranges. The *P* values were measured using the Mann Whitney *U* test (B, D, F, I, K, M, O, P). Data are presented as median and interquartile ranges (IQR) except for standard deviation (SD) for the plot G.

To better characterize the migratory capacity of the CD8^+^CD226^+^ T cell subpopulation, we subjected them to further analysis for the expression of various homing receptors. We found that CD8^+^CD226^+^ T cells had a significantly higher frequency of skin homing receptors, such as CCR4 and CLA [50, 51] (Fig. 5A–D). Given that RUNX2 expression is essential for the development of cytotoxic epidermal tissue‐resident memory T cells [52], we investigated the expression of this marker in CD8^+^CD226^+^ T cells compared to their negative counterparts. Interestingly, we observed significantly higher expression of RUNX2, but not RUNX3, in CD8^+^CD226^+^ T cells (Fig. 5E,F). This was further confirmed at the transcriptional level (Fig. 5G). In contrast, CD226^−^ subsets were significantly enriched with the gut‐homing receptor β7 integrin [53, 54], while there was no significant difference between the frequency of CCR6‐expressing cells among CD226^+^ and CD226^−^ T cells (Fig. 5H–K). It is worth mentioning that we also did not find any significant difference in the frequency of T cells expressing CCR7 among CD226^+^ and CD226^−^CD8^+^ T cells in CLL patients despite a trend towards a reduction among CD226^+^ T cells (Fig. S4M,N). To further evaluate the migratory capacity of CD226^+^ and CD226^−^ T cells, we performed a migration assay, which confirmed a significantly higher migratory capacity in CD226^+^ T cells versus their negative counterparts in response to CCL‐17, but not to FBS, *in vitro* (Fig. 5L,M and Fig. S2A).

Finally, to assess the survival capacity of these CD8^+^ T cell subsets, we analyzed KLRG1 and CD127 (IL‐7R) expression, as long‐lived T cells typically express higher levels of CD127 but lower levels of KLRG1 [55, 56]. Our observations revealed that CD8^+^CD226^+^ T cells in CLL patients were enriched with CD127 but not KLRG1‐expressing cells (Fig. 5N–P). These findings suggest that CD8^+^CD226^+^ T cells exhibit a greater longevity and migratory capacity compared to their negative counterparts.

### 3.6 Differential levels of cytokines and chemokines in the plasma of CLL patients

**Fig. 6:**
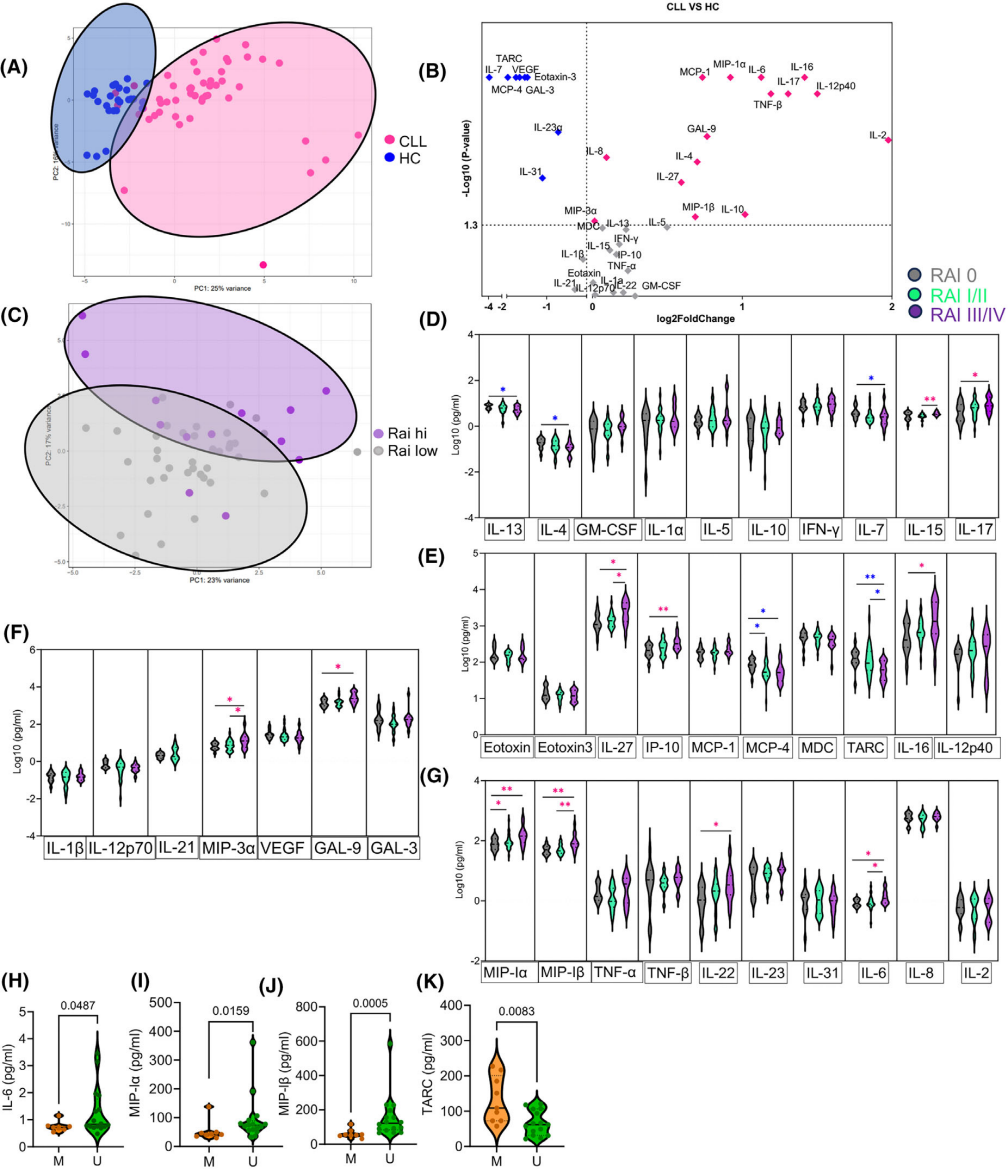
Differential plasma cytokines/chemokines levels in chronic lymphocytic leukemia (CLL) versus healthy controls (HCs). (A) Principal Component Analysis (PCA) on the Euclidean distances calculated between the cytokine and chemokine profiles of plasma samples obtained from CLL patients and HCs. (B) The volcano plot illustrating the magnitude and significance of differences in cytokines/chemokines concentrations (measured by the Mesoplex assay) in patients with CLL versus HCs. (C) PCA on the Euclidean distances derived from the cytokines and chemokines profiles of CLL patients between those with RAI stage III/IV and lower clinical stages. (D–G) Normalized and calculated concentrations of cytokines and chemokines in the plasma of CLL patients with low (0), intermediate (I/II), and high (III/IV) Rai stages. (H–K) Cumulative data comparing IL‐6, MIP‐1α, MIP‐1β, and TARC concentrations in the plasma of patients with CLL patients with mutated (M) and unmutated (U) immunoglobulin heavy chain (IgHV) status, respectively. The cumulative data are shown as the medians and interquartile ranges. The *P* values were measured using the Mann Whitney *U* test (B, H–K) or Kruskal–Wallis test followed by Dunn's *post‐hoc* tests (D–G). *P < 0.05, **P < 0.01.

Considering that cytokines and chemokines can modulate T cell effector functions [45, 57], we quantified a wide range of cytokines and chemokines in the plasma of 55 CLL patients and 25 HCs. Principal component analysis (PCA) based on Euclidean distances separated HCs from CLL patients in a two‐dimensional plot (Fig. 6A). Specifically, we found that TNF‐β, IL‐16, MIP‐1α, MIP‐1β, IL‐12/23p40, IL‐10, IL‐8, MCP‐1, IL‐17A, IL‐2, IL‐6, IL‐27, GAL‐9, IL‐4, and MIP‐3α were significantly elevated in CLL patients compared to HC (Fig. 6B and Fig. S2B). In contrast, Eotaxin‐3, IL‐31, IL‐23α, Gal‐3, IL‐7, TARC, VEGF, and MCP‐4 (CCL13) were significantly lower in CLL patients (Fig. 6B). Among elevated cytokines in CLL patients, IL‐16, IL‐6, MIP‐1α (CCL3), and MCP‐1 showed the highest deviation compared to HCs (*P* < 0.0001), followed by IL‐17A, TNF‐β, IL‐12/23p40, and IL‐2 (Fig. 6B).

**Fig. 7:**
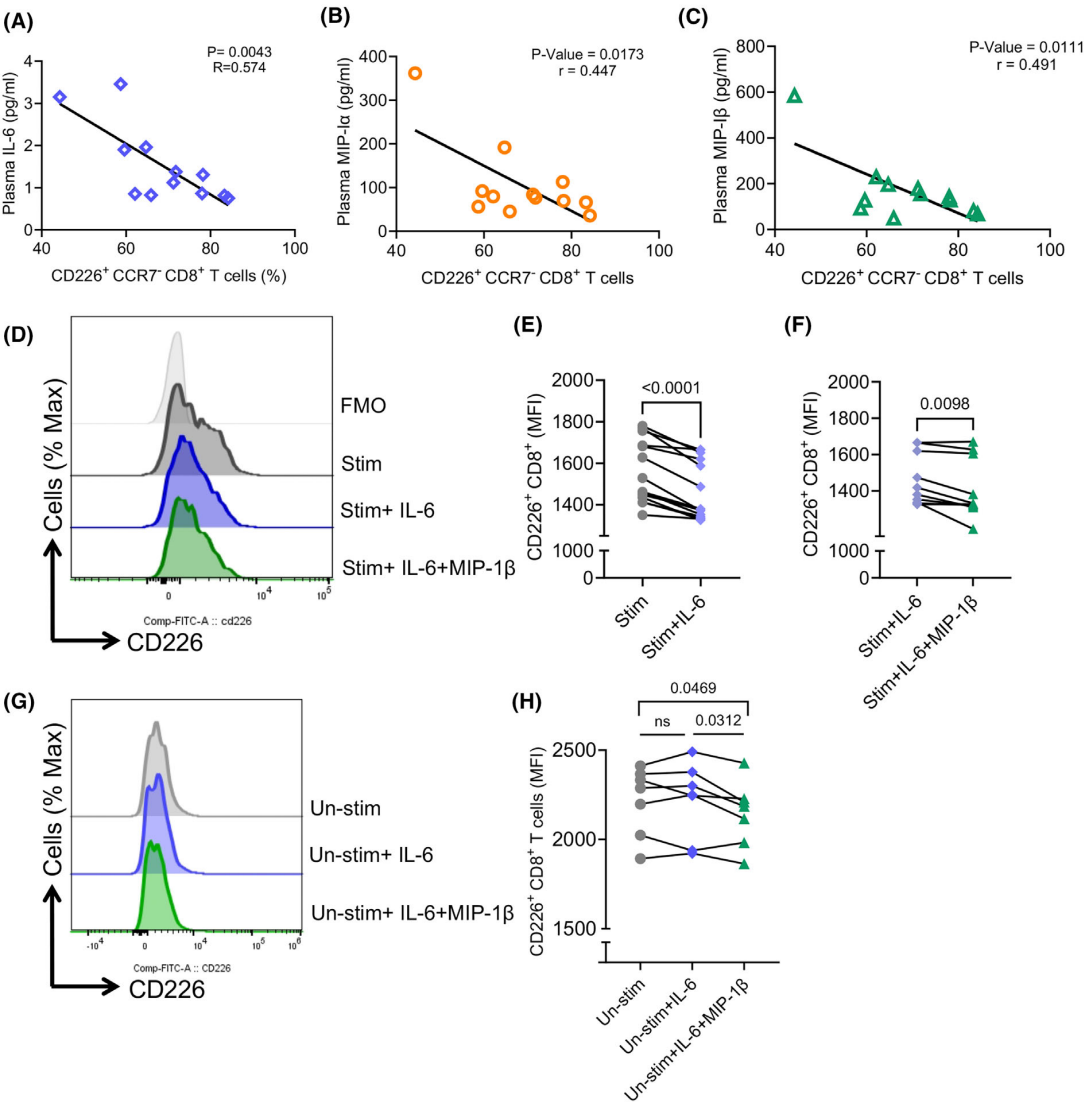
Elevated levels of IL‐6 and MIP‐1β contribute to the reduction of CD226 expression in CD8^+^ T cells. (A) Scatter plot of the correlation between percentages of CD8^+^CD226^+^ effector T cells in peripheral blood mononuclear cells (PBMCs) with the IL‐6, (B) MIP‐1α, and (C) MIP‐1β concentrations in the plasma of chronic lymphocytic leukemia (CLL) patients. (D) Representative histogram plots, and (E) cumulative data of the mean fluorescence intensity (MFI) of CD226^+^ in CD8^+^ T cells following stimulation with anti‐CD3/CD28 in the presence or absence of recombinant human IL‐6 (0.02 μg·mL^−1^) (F) MIP‐1β (1 ng·mL^−1^). (G) Representative histogram plots, and (H) cumulative data of the MFI of CD226^+^ in CD8^+^ T cells following treatment with recombinant human IL‐6 (0.02 μg·mL^−1^) or IL‐6 plus MIP‐1β (1 ng·mL^−1^) in the absence of anti‐CD3/CD28 for 2 days. The *P*‐values were measured using linear regression (A–C) and the Wilcoxon test (E, F, H).

To determine whether the Rai stage influences the cytokine levels in the plasma of CLL patients, we performed additional analysis. PCA to some degree separated CLL patients with higher Rai stage from those with lower Rai stages (Fig. 6C). Specifically, we found the plasma levels of IL‐27, IL‐6, MIP‐1α, MIP‐1β, MIP‐3α, IL‐15, IL‐17, IL‐16, IL‐22, Gal‐9, and IP‐10 were significantly elevated in CLL patients with higher Rai stage (III/IV) than in those with intermediate (I/II) or low Rai (0) stages (Fig. 6D–G). Notably, IL‐27, IL‐6, MIP‐Iβ, and MIP‐3α exhibited pronounced elevation in higher Rai stages relative to both intermediate and lower Rai stages, mirroring the pattern observed in CD8^+^CD226 EFF and EM T‐cell percentages (Fig. 6D–G). In contrast, we found that IL‐4, IL‐13, IL‐7, TARC, and MCP‐4 were significantly reduced in CLL patients with higher Rai stage (III/IV) than in those with intermediate (I/II) or lower Rai (0) stages (Fig. 6D–G). Additionally, we compared the cytokine profile in mutated versus unmutated CLL patients, which showed the clustering of the majority of the mutated group (Fig. S2C). Specifically, patients with mutated CLL exhibited a significant elevation in IL‐6, MIP‐1α, and MIP‐1β but a reduction in TARC levels compared to their unmutated counterparts (Fig. 6H–K). Notably, we found that MIP‐1α, IL‐6, and MIP‐1β concentrations were inversely correlated with the percentages of CD8^+^CD226^+^ effector T cells, specifically in patients with higher RAI stages (Fig. 7A–C).

Therefore, to determine whether these cytokines/chemokines contribute to the reduction in CD226^+^ expression in CLL, we treated stimulated T cells (anti‐CD3/CD28) with IL‐6, MIP‐1β, and MIP‐1α for 2 days. We found that IL‐6 significantly reduced the surface expression of CD226 (Fig. 7D,E) and MIP‐1β exhibited a synergistic effect with IL‐6 (Fig. 7D,F). However, this was not the case for MIP‐1α (Fig. S2D). Our additional studies, aimed at determine the effects of these cytokines in the absence of T cell activation, revealed that only a combination of IL‐6^+^MIP‐1β, but not IL‐6 alone, reduces the intensity of CD226^+^ T cells (Fig. 7G,H). Finally, to determine whether the reduction in CD226‐expressing‐cells is related to the shedding of this co‐stimulatory molecule, we quantified soluble CD226 concentrations in the plasma of CLL patients across varying Rai stages. However, our analysis revealed no significant differences in CD226 concentrations across different Rai staging categories among CLL patients (Fig. S2E).

### 3.7 Differential gene expression profile in CD226^+^ compared with CD226^−^CD8^+^ T cells

**Fig. 8:**
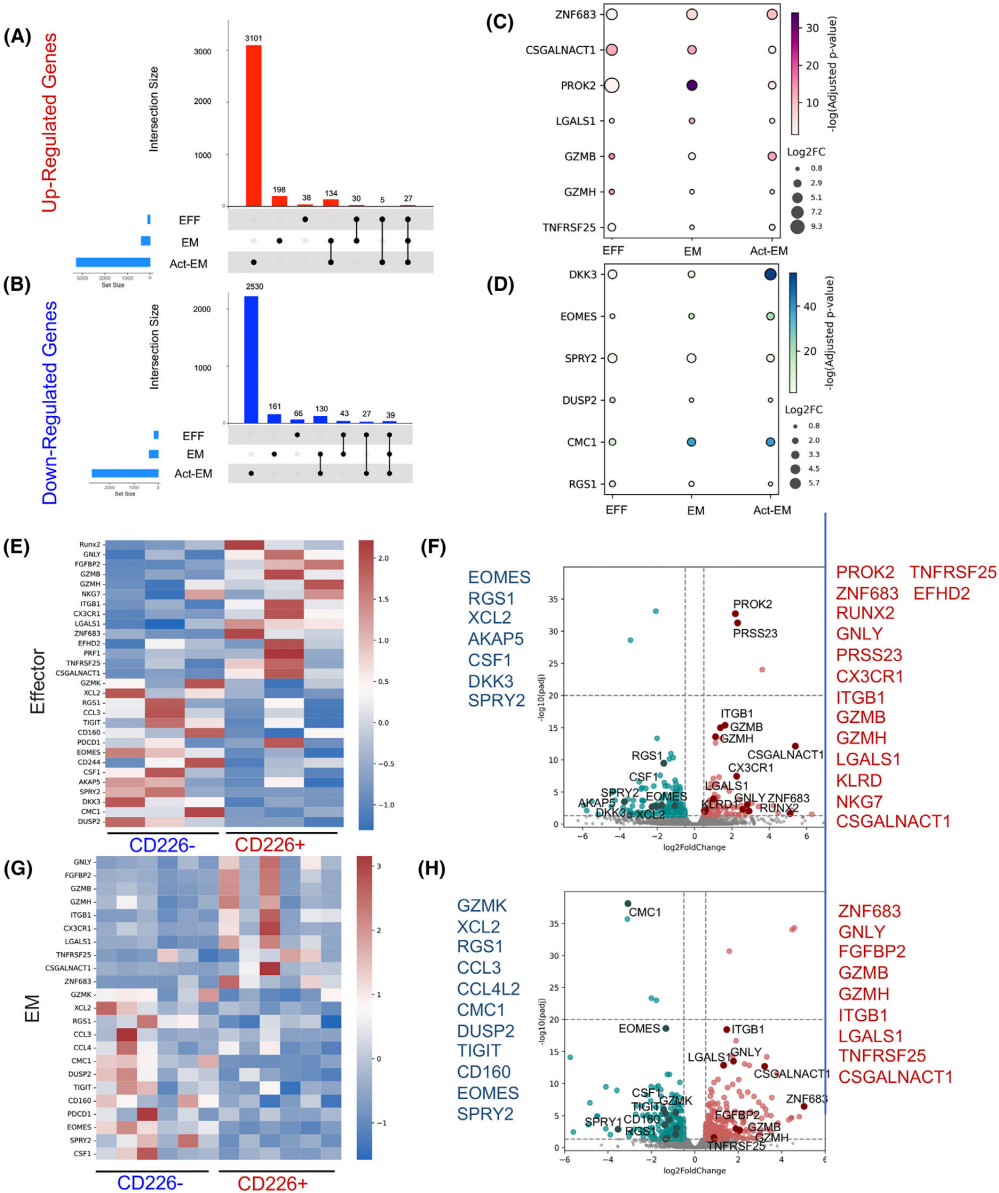
Differential gene expression profile in CD8^+^CD226^+^ compared with CD8^+^CD226^−^ T cells. (A) UpSet plots depicting transcripts upregulated and (B) downregulated in CD226^+^CD8^+^ T cells compared to CD226^−^CD8^+^ T cells across three subsets: resting effector memory (EM), effector (EFF), and T cell receptor (TCR)‐activated EM. The sidebars represent the number of differentially expressed (DE) transcripts in each comparison. Vertical bars represent the intersection size, indicating the number of transcripts that are DE in one or more comparisons. Black dots indicate the specific comparisons in which these transcripts are DE. (C) Bubble plots depicting the highly upregulated and (D) downregulated transcripts common to all three subsets: EM, EFF, and TCR‐activated EM. (E) Heatmaps and (F) volcano plots depicting differentially expressed transcripts related to EFF CD8^+^ T cell genes in CD226^+^ versus CD226^−^ cells. (G) Heatmaps and (H) volcano plots depicting differentially expressed transcripts related to EM CD8^+^ T cell genes in CD226^+^ versus CD226^−^CD8^+^ T cells.

To verify our observations and better understand the properties of CD226^−^ and CD226^+^CD8^+^ T cells, we reanalyzed a publicly available bulk RNA‐seq dataset that was performed across three subsets of resting EM, EFF, and TCR‐activated EM CD8^+^ T cells [28]. For our analysis, a transcript was considered differentially expressed (DE) if it had an FDR < 0.05, with a minimum log_2_ fold change (Log_2_FC) less than −0.5 or greater than +0.5. Comparative analysis among these three subsets confirmed that 27 genes were consistently upregulated (Fig. 8A), while 37 genes were downregulated in CD226^+^ T cells (Fig. 8B).

Among the highly upregulated genes in CD8^+^CD226^+^ T cells across all three subsets, we observed those associated with cytotoxicity, including *GzmB*, *GzmH*, and *LGALS1* (Fig. 8C). These cytotoxic genes are linked to non‐progressive multiple myeloma compared with rapidly progressive cases [22, 58]. Other genes (e.g., *PROK2* and *csgalnact1*) related to effector functions and cytotoxicity, which are highly upregulated in effector T and NKT‐like cells [59, 60], were upregulated in CD226^+^ cells. Remarkably, among the highly expressed genes within CD8^+^CD226^+^ T cells, *ZNF683* stood out (Fig. 8C), as high expression of *ZNF683* in CD8^+^ T cells indicated their derivation from stem‐like memory cells and distinctiveness from terminally exhausted cells [21, 61]. Interestingly, the expression of *ZNF683* in CD8^+^ T cells, a pivotal regulator of T cell activation and cytotoxicity, has been reported to correlate with a better response to anti‐PD‐1 therapy in patients with CLL and Richter Syndrome [21, 61].

In contrast, genes significantly upregulated in CD226^−^CD8^+^ T cells across all subsets were associated with immunosuppression and exhaustion (Fig. 8D). In particular, *RGS‐1*, *DUSP2*, and *CMC1* are associated with immunosuppression and exhaustion as shown in CD8^+^ T cells of patients with rapidly progressive multiple myeloma [22, 62, 63]. *EOMES* was also highly elevated in CD8^+^CD226^−^ T cells, confirming our observations at the protein level (Fig. 4F,G). *DKK3* is an immunomodulatory gene that participates in the hyporesponsiveness of CD8^+^ T Cells [64] and was significantly upregulated in CD226^−^CD8^+^ T cells. In addition, *SPRY‐1*, a negative regulator of effector and memory CD8^+^ T cell formation [65], was highly upregulated in CD226^−^CD8^+^ T cells (Fig. 8D).

Moreover, we conducted further analysis of these subsets, which revealed distinct transcriptional markers that distinguish CD226^+^ from CD226^−^CD8^+^ within both the EM and EFF subsets (Fig. 8E–H). More importantly, both EFF and EM CD8^+^CD226^+^ T cells exhibited significant upregulation of genes associated with cytotoxicity (Fig. 8E–H). As outlined above, important genes in this upregulated profile included *GzmB*, *GzmH, LGALS1*, *ITGB1*, *GNLY*, *EFHD2*, and *NKG7*. In contrast, we observed marked downregulation of genes related to immunomodulation and exhaustion in both EFF and EM CD8^+^CD226^+^ T cells (Fig. 8H). The most down‐regulated genes were *XCL2*, *RGS1*, *CCL3*, *CCL4L2*, *CMC1*, *DUSP2*, *TIGIT*, *CD160*, and *GzmK*. Notably, the presence of these genes has been linked to a less favorable prognosis in multiple myeloma [22, 58].

### 3.8 Transcriptional factors (TFs) analyses revealed higher effector functions in CD8^+^CD226^+^ T cells

To better characterize CD226^+^ versus their negative counterparts, we evaluated their TFs using decoupleR. These analyses revealed the upregulation of TFs, including *MYC*, *E2F1*, *E2F2*, *E2F3*, *E2F4*, and *FOXM1* (Fig. S3), which are linked to a higher proliferative capacity and response to stimulation [66]. Similarly, the upregulation of *JUN*, *JUNB*, *AP1*, *NFATC2*, and *NFKB2* demonstrates (Fig. S3) that CD8^+^CD226^+^ T cells are highly activated and potentially producing inflammatory cytokines [67]. Additionally, *STAT3*, and *STAT5B* are associated with T cell metabolism, cytokine signaling, and survival [68], with their upregulation further reinforcing that these cells are likely in an active and proliferative state. The upregulation of Tcf1 (encoded by *Tcf7*), which is associated with stem‐like CD8^+^ T cells, further supports this observation [69]. Moreover, the upregulation of *CREB*‐binding protein, which is involved in chromatin remodeling and transcriptional activation, suggests active gene expression and response to stimulation, which is crucial in TCR‐dependent *TNF‐α* gene expression [70].

Finally, *DNMT1*, a DNA methylation TF, and *ID1*, a regulator of cell differentiation, were upregulated in CD8^+^CD226^+^ T cells [71, 72]. In contrast, the downregulation of *FOXO3*, *AIRE*, and *RFXANK* refutes the regulatory functions of these cells and supports their highly activated status [73, 74]. For example, the *IKZF1* gene has been implicated in regulating CD8^+^ T cell differentiation by restricting autocrine IL‐2 production, and its downregulation in CD226^+^ T cells may enable them to differentiate into memory or effector subsets [75]. Additionally, we found the downregulation of *CIITA*, whose critical role of *CIITA* in MHCII antigen presentation is well‐documented [76], although its role in CD8^+^ T cells remain unclear. The downregulation of *RB1* and *TP53* further supports the idea that these cells are less likely to undergo cell death [77] and may proliferate unchecked for longer periods. Other important downregulated TFs, such as *REST*, *HBP1*, and *HDAC4* (Fig. S3), which are associated with quiescence and immunosuppression, reinforce the idea that these cells are highly activated and no longer in a resting state [78, 79]. Finally, downregulation of *ETV3* and *RUNX3* may indicate a loss of regulatory properties in these cells, given their role in regulating T cell exhaustion [80].

## 4 Discussion

CD8^+^ T cells play an essential role in eliminating tumor cells [10], and their cytotoxic capabilities have been determining the progression of various solid and hematological malignancies [11, 22, 58]. In this study, we investigated the frequency and functionality of CD8^+^CD226^+^ T cells in CLL patients. The notable observation was that these highly cytotoxic and functional T cells were significantly decreased in advanced Rai stages and unmutated IgVH patients.

Upon further analysis across different Rai stages, we observed that CLL patients with Rai stage IV/III had a significantly lower frequency of CD226‐expressing CD8^+^ T cells compared to Rai stages I/II and Rai stage 0 in their blood circulation. This reduction was more pronounced within the EFF and EM CD8^+^ T cell subsets, with the CD226‐expressing subset being significantly lower in those with Rai Stage IV/III than in those with Rai stages I/II and Rai Stage 0. Considering that EFF and EM T cells are highly antigen‐experienced [20], their reduction can have important clinical implications. Interestingly, we found significantly lower percentages of CD8^+^CD226^+^ T cells in unmutated IgVH, which is associated with worse prognosis [81]. This phenomenon was mainly pronounced within the EFF and EM subsets but not in naïve and CM CD8^+^ T cells.

Notably, we observed a differential distribution of CD226^+^ cells among different subsets of CD8^+^ T cells between the HCs and CLL patients. For example, we found that CD8^+^CD226^+^ T cells were distributed among EM, CM, and EFF CD8^+^ T cells in HCs, as previously reported [24]. In contrast, CD226^+^ cells were mainly populated in the CM and EM compared to the EFF and naive subpopulations in CLL patients.

Further investigations to characterize the functionality of CD8^+^CD226^+^ T cells compared to their negative counterparts revealed that these cells exhibit profound effector functions, including increased cytokine and cytolytic molecule expression, degranulation and proliferation capacities, and migration ability compared to their negative counterparts, as reported by other groups [25, 28, 82]. Considering that cytotoxicity is coupled with proliferative capacity, our observations support the polyfunctionality of CD8^+^CD226^+^ T cells [35]. Additionally, we found that CD226^+^ T cells were enriched with NK‐T‐like cells, with prominent cytotoxic capabilities [83], and have been linked to a better clinical outcome in CLL [84, 85], multiple myeloma [86], and colorectal cancer [87]. Interestingly, we found that CD8^+^CD226^+^ T cells, especially EM and EFF subsets, were highly enriched with T cells expressing CD56, a marker of NK‐T‐like cells. Furthermore, we found that effector CD8^+^CD226^+^ T cells expressed higher levels of CD29, which identifies effective IFN‐γ‐producing and cytotoxic CD8^+^ T cells [88].

Overall, these observations support the notion that despite their heterogeneity, CD226^+^CD8^+^ T cells exhibit robust effector functions. This hypothesis is further supported by the upregulation of TFs, such as cell cycle regulators like *MYC*, *E2Fs*, and *FOXM* [66]. This aligns with TFs associated with T cell activation, including *JUN*, *STAT3*, *AP1*, and *CREB*‐binding protein [67, 70]. Additionally, the upregulation of *Tcf7* suggests potential self‐renewing capacity [4] and supports their stem‐like properties, allowing them to retain long‐term memory potential with robust proliferative capacity, as reported for Tcf1^+^CD8^+^ T cells upon anti‐PD‐1 therapy [89]. Furthermore, *DNMT1*, a DNA methylation TF, and *ID1*, a regulator of cell differentiation, are upregulated in CD8^+^CD226^+^ T cells, which could indicate epigenetic reprogramming. This may allow these T cells to adopt a more effector‐like or proliferative phenotype [71, 72].

In contrast, the downregulation of *FOXO3*, *AIRE*, and *RFXANK* contradicts their regulatory functions, suggesting instead a highly activated status for these cells [73, 74]. For example, the *IKZF1* gene has been implicated in regulating CD8^+^ T cells differentiation by restricting autocrine IL‐2 production, and its downregulation in CD226^+^ T cells may enable their differentiation into memory or effector subsets [75]. Overall, TFs suggest that CD8^+^CD226^+^ T cells exhibit a highly proliferative, activated, and likely effector‐like state, with diminished regulatory and quiescent properties.

In contrast, we found that CD226^−^CD8^+^ T cells express elevated levels of co‐inhibitory receptors (e.g., CD160, TIGIT, 2B4, CD244, and PD‐1) and Eomes, consistent with another report [24]. These findings suggest that CD226^−^CD8^+^ T cells exhibit a dysfunctional phenotype, as indicated by their lower proliferative capacity, cytokine expression, and cytotoxicity.

Another interesting observation is that CD8^+^CD226^+^ T cells express elevated levels of the chemokine receptors CLA, CCR4, and RUNX2, which contribute to T‐cell trafficking into the skin. This capability suggests that CD8^+^CD226^+^ T cells may protect against the most common secondary tumors in CLL patients [38, 90]. Our results may, in part, highlight the role of RUNX2 in EFF CD8^+^CD226^+^ immunosurveillance at the epithelial barriers, as previously reported in melanoma [52]. Therefore, the loss of CD8^+^CD226^+^ T cells might be a potential explanation for the poorer clinical outcomes of secondary skin cancer in CLL patients with a higher Rai stage [91, 92]. However, further studies are needed to investigate the effector functions of RUNX2‐expressing CD226^+^CD8^+^ T cells in CLL and their potential to migrate to the skin.

Considering that the presence of soluble biomarkers, such as cytokines, chemokines, and galectins, in the plasma may modulate T cell effector functions [38, 93, 94], we quantified their concentrations in our cohorts. We found elevated levels of IL‐2, IL‐10, TNF‐β, IL‐17, IL‐12p40, IL‐8, MIP‐1α, MIP‐1β, and MCP in our patients with CLL, as reported in another cohort [95]. In agreement with other studies, we observed a significant increase in IL‐16 and Gal‐9 levels in the plasma of patients with CLL [38, 45, 96]. Although Gal‐9 levels were elevated in the plasma of our CLL cohort, Gal‐3 levels declined significantly. The role of Gal‐3 in solid and hematological cancers has been a subject of debate. For example, elevated expression of Gal‐3 in the bone marrow is an independent unfavorable prognostic factor in acute myeloid leukemia [97]. Nevertheless, the role of Gal‐3 in CLL has not been well‐defined, aside from an inconclusive report on the expression of Gal‐3 mRNA in leukemic B cells [98]. While our findings show a significant decline in plasma Gal‐3 levels in CLL patients compared to HCs, this reduction was not associated with disease stage. Therefore, future studies should investigate the role of Gal‐3 in the BM of CLL patients. Among the elevated cytokines, IL‐10, IL‐6, MIP‐1α, MIP‐1β, MCP‐1, IL‐8, and IL‐4 have been reported to promote the survival of neoplastic B cells by supporting various antiapoptotic mechanisms [99, 100, 101, 102].

We found that IL‐6, MIP‐1β, and MIP‐1α were elevated in both late Rai stages and in patients with unmutated IgVH, which resembles an opposite pattern to the frequency of the CD226‐expressing CD8^+^ T cell subset. This observation prompted us to investigate the potential link between these soluble factors and the frequency of CD8^+^CD226^+^ T cells. Interestingly, we found a moderate inverse correlation between IL‐6, MIP‐1β, MIP‐1α and the percentage of EFF CD8^+^CD226^+^ T cells. To better understand the underlying mechanism, we cultured PBMCs in the presence of these soluble factors and observed that, as previously reported, IL‐6 downregulates the expression of CD226 in CD8^+^ T cells [103], and this effect was further pronounced in the presence of MIP‐1β. These adverse effects on CD226^+^ T cells may partially explain the reported role of IL‐6 in the induction of dysfunctional CD8^+^ T cells and downregulation of co‐stimulatory, coupled with the upregulation of co‐inhibitory receptors via STAT‐3 [103, 104]. Similarly, MIP‐1β has been implicated in impaired CD8^+^ T cells in patients with NSCLC [105]. Therefore, our results provide further insights into the potential role of IL‐6 and MIP‐1β in CD8^+^ T cell dysfunction.

While the higher TIGIT/CD226 ratio on CLL cells is reported to be typical of a more indolent disease [106], there are no other reports on the frequency of CD8^+^CD226^+^ T cells in CLL patients. Therefore, this is the first study, to our knowledge, to characterize human CD8^+^CD226^+^ T cells in terms of phenotypic and functional properties in CLL patients.

Our study has several limitations. First, our cohort was small and single‐center; therefore, more studies with diverse cohorts are needed. Additionally, since the mean age of patients with CLL is 70 years, our results may not be generalizable to all age groups. While we are aware that T cells in blood circulation differ significantly from those derived from lymph nodes, biopsies from lymph nodes are not commonly performed in CLL patients. This limited the scope of our study to blood‐based analysis. Furthermore, we could not obtain a larger blood sample size to conduct more in‐depth analyses, such as RNAseq or single‐cell RNAseq, on different CD226^+^ and CD226^−^CD8^+^ T cell subsets from CLL patients. Such analyses would be instrumental in identifying changes in TFs and genes associated with the reduced survival of CD226^+^ cells, which could inform potential therapeutic interventions. In addition, our study did not include a pre‐ and post‐treatment cohort, limiting our ability to assess the impact of CLL treatment on CD8^+^CD226^+^ T cells. Due to the low number of CD8^+^ T cells in CLL patients, we could not assess the cytotoxic capabilities of CD226^+^ versus CD226^−^ T cells against CLL cells. Further studies are needed to determine the cytotoxicity of CD8^+^CD226^+^ T cells against CLL cells. Additionally, our characterization of CLL patients was limited by the lack of more modern prognostic tools (e.g., CLL‐IPI) and the absence of TP53 mutation status in our cohort. Another important study to consider in the future will be assessing the migratory capacity of CD8^+^CD226^+^ T cells to the skin and whether they contribute to the prevention of secondary skin tumors. Future studies with broader access and a wider range of data collection could address these limitations and further our understanding of this T cell subset in CLL.

Taken together, we found that CD8^+^CD226^+^ T cells were decreased in high‐risk CLL patients, and this observation was more prominent in the EM and EFF subsets. Second, our observations revealed that CD8^+^CD226^+^ T cells are highly polyfunctional and exhibit greater cytokine and cytolytic molecule expression, proliferative capacity, degranulation, and migration capacity. Third, our observations suggest that higher levels of IL‐6, accompanied by MIP‐1β, may contribute to the downregulation of CD226 in CLL patients, which is associated with poor prognosis. Finally, our findings highlight the potential role of RUNX2 in CD8^+^CD226^+^ T cell effector functions positively correlated with tumor‐infiltrating CD8^+^ T cells [52]. However, further studies are needed to determine whether the preservation of CD8^+^CD226^+^ T cells alters the course of CLL disease and/or reduces secondary skin tumors. If this is the case, targeting IL‐6 and MIP‐1β to preserve polyfunctional CD8^+^CD226^+^ T cells should be considered a potential therapeutic strategy.

## 5 Conclusion

Our study highlights the reduction of CD8^+^CD226^+^ T cells in CLL patients, particularly in advanced Rai stages and unmutated IgVH cases, which correlates with a poor clinical prognosis. The decreased frequency of CD8^+^CD226^+^ T cells, especially within the EFF and EM subsets, suggests compromised cytotoxic potential and impaired immune surveillance. Furthermore, the upregulation of effector‐related transcription factors and a distinct expression profile of chemokine receptors in CD226^+^ T cells indicate their robust effector functions and potential to contribute to improved outcomes in other cancers. Elevated cytokine levels, particularly IL‐6 and MIP‐1β, were inversely correlated with CD226^+^ T cell frequency, suggesting that these soluble factors may contribute to CD8^+^ T cell dysfunction in CLL. These findings provide important insights into the role of CD8^+^CD226^+^ T cells in CLL pathogenesis and highlight their potential as biomarkers or therapeutic targets.

## Conflict of interest

The authors declare no conflict of interest.

## Author contributions

MR designed and performed all experiments, analyzed the data, designed the figures, and wrote the first draft of the manuscript. SS assisted with the experimental design and RNA‐seq analysis. ACP as a hemato‐oncologist, provided most of the blood samples and advised and assisted with the clinical data analysis. SBG provided some samples and advised on the study. SE conceptualized the study, assisted in the experimental design, secured funds and resources, supervised the research, and wrote the manuscript.

### Peer review

The peer review history for this article is available at https://www.webofscience.com/api/gateway/wos/peer‐review/10.1002/1878‐0261.13793.

## Supporting information


**Fig. S1.** The gating strategy on peripheral blood mononuclear cells (PBMCs) for CD8^+^CD226^+^ T cells, their frequency among different CD8^+^ T cell subsets in healthy controls, and the expression of CD29 and co‐inhibitory receptors in CD226^+^/CD226^−^CD8^+^ T cells.
**Fig. S2.** Principal component analysis (PCA) of cytokines in mutated and unmutated immunoglobulin heavy chain (IgHV) chronic lymphocytic leukemia patients (CLL) and soluble CD226 levels in the plasma of CLL patients in various Rai stages.
**Fig. S3.** Representing upregulated and downregulated transcriptional factors (TFs) in CD226^+^ versus their negative counterparts using decoupleR.

**Table S1.** Clinical and demographic information of our study cohorts.

## Data Availability

The datasets used or analyzed in this study are included in the main article and Supporting Information. The RNA‐Seq databases (GSE133397 and GSE135291) are publicly available [28].
