# Supplementary material for: Polyfunctional CD8+ CD226+ RUNX2hi effector T cells are diminished in advanced stages of chronic lymphocytic leukemia
Source: Mol Oncol. 2025 Jan 7;19(5):1347–70. doi: 10.1002/1878-0261.13793 (PMC12077284; doi:10.1002/1878-0261.13793)
Supplement: Supplementary file 1 — Fig. S1. The gating strategy on peripheral blood mononuclear cells (PBMCs) for CD8+CD226+ T cells, their frequency among different CD8+ T cell subsets in healthy controls, and the expression of CD29 and co‐inhibitory receptors in CD226+/CD226−CD8+ T cells. Fig. S2. Principal component analysis (PCA) of cytokines in mutated and unmutated immunoglobulin heavy chain (IgHV) chronic lymphocytic leukemia patients (CLL) and soluble CD226 levels in the plasma of CLL patients in various Rai stages. Fig. S3. Representing upregulated and downregulated transcriptional factors (TFs) in CD226+ versus their negative counterparts using decoupleR. [file MOL2-19-1347-s001.pdf]

Supplemental Fig. 1

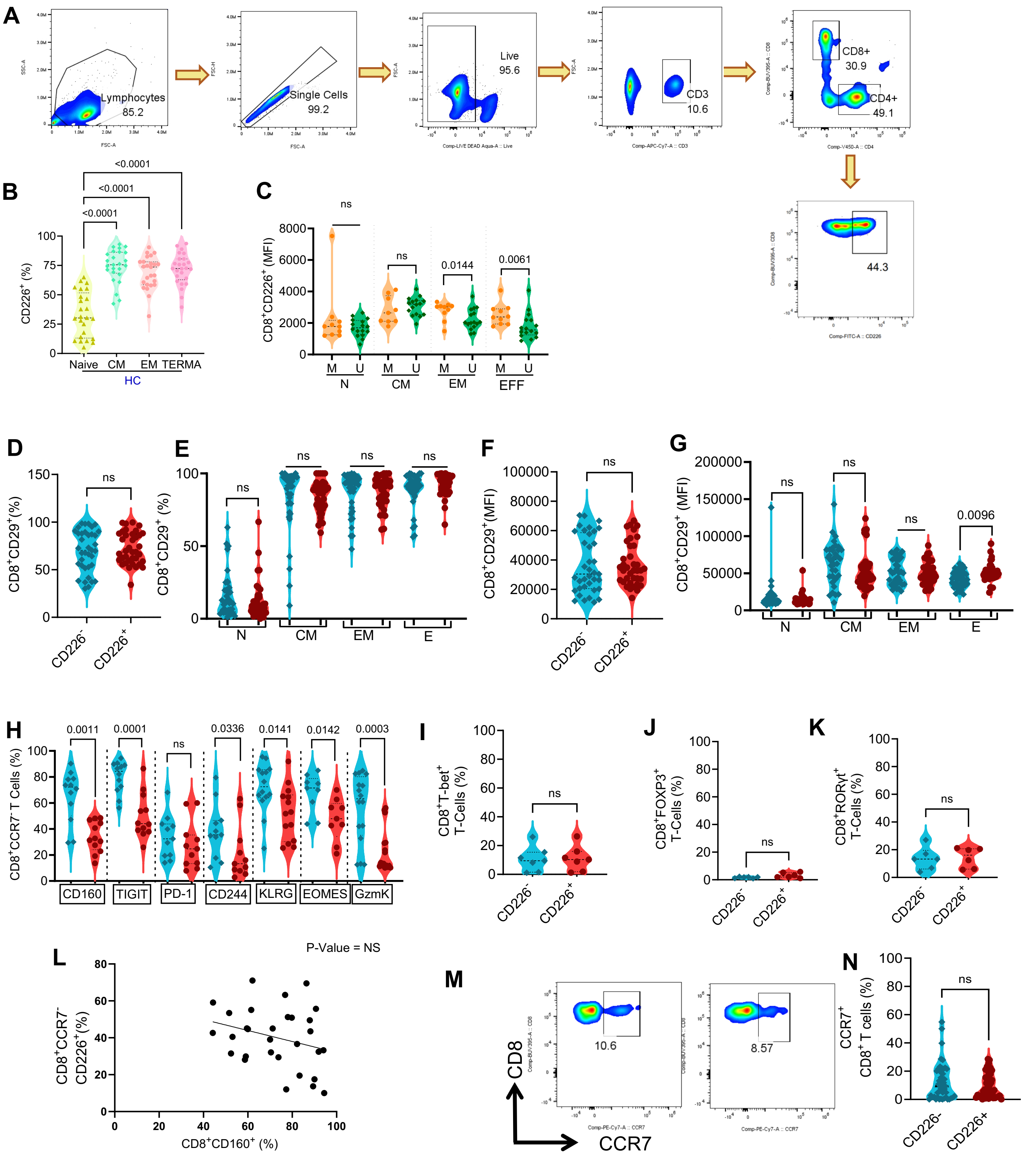

Supplemental Fig. 2

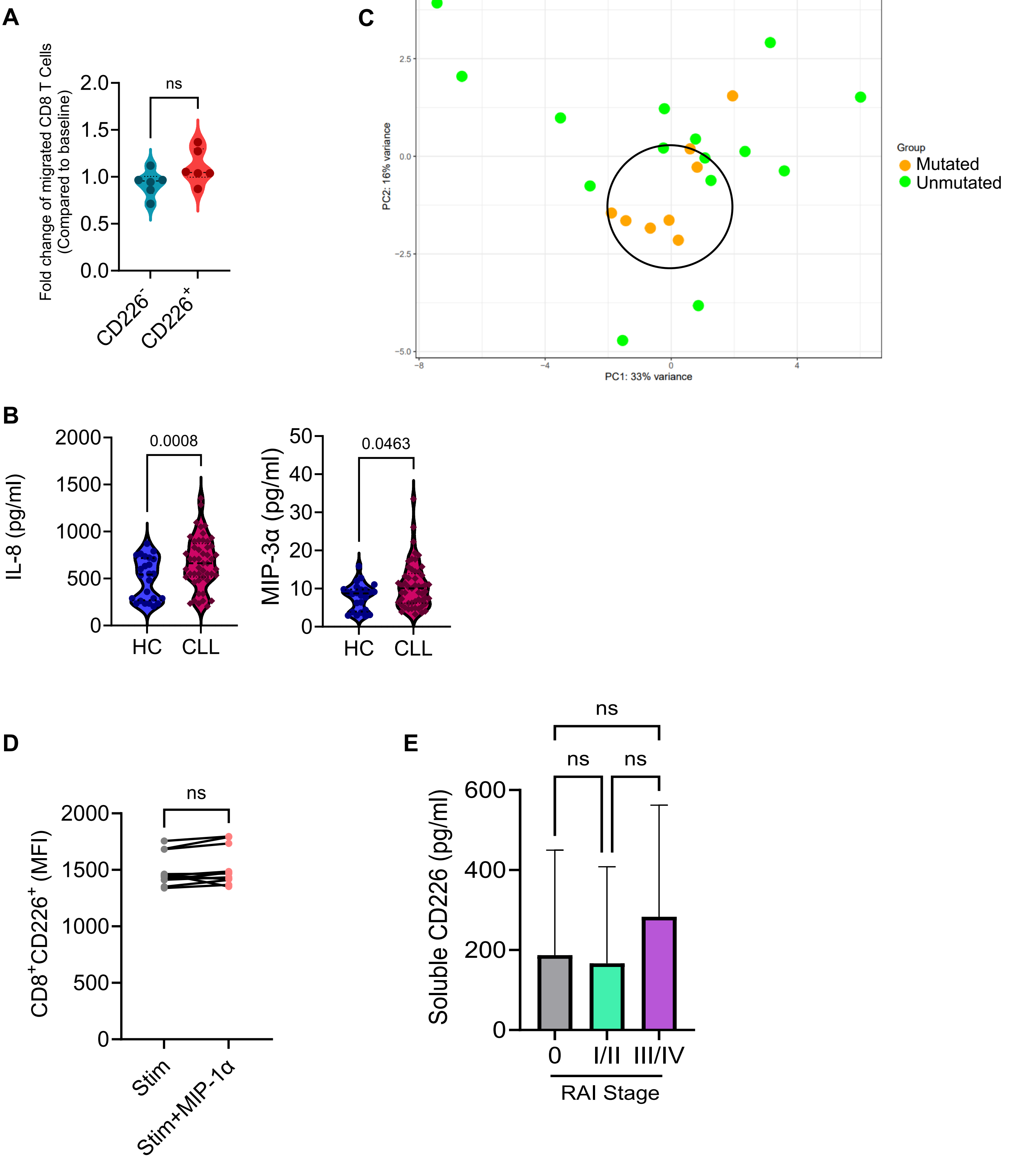

Supplemental Fig. 3

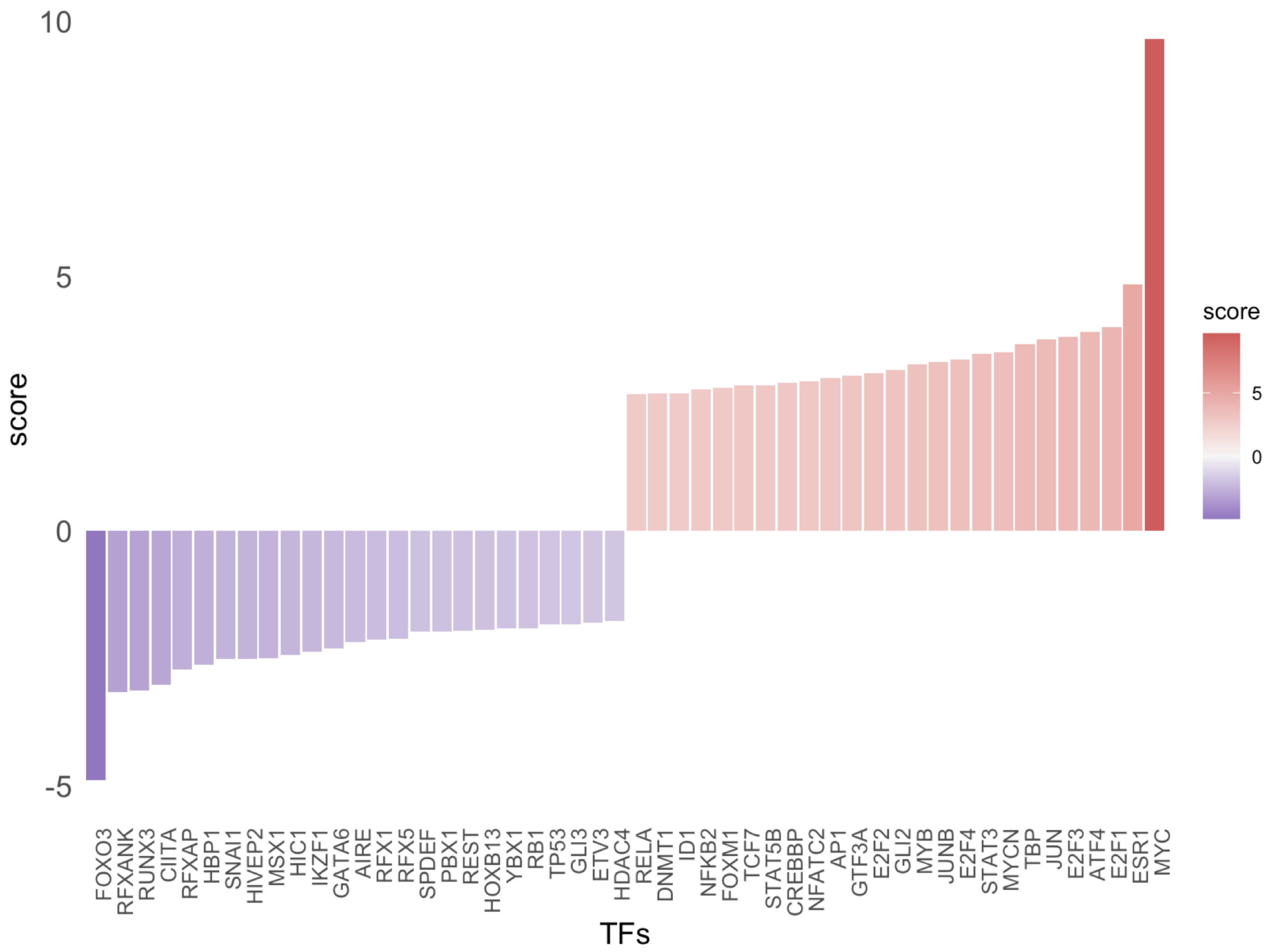

Supplemental Figure legends

S Figure 1. (A) Representative flow plots of the gating strategy for CD226+CD8+ T cells. **(B)** Cumulative data comparing the frequency of CD226 expressing cells among different subsets of CD8+ T cells in HCs. **(C)** Cumulative data of the mean fluorescence intensity (MFI) of CD226 in naïve (N), central memory (CM), effector memory (EM), and effector (EFF) CD8+ T cells in patients with CLL with mutated and unmutated IgHV mutational status. **(D)** Cumulative data showing the frequency of CD29 among CD226+ versus CD226- CD8 T cells. **(E)** Cumulative data showing the frequency of CD29 among different subsets of CD226+ and CD226-CD8+ T cells in CLL patients. **(F)** Cumulative data showing the MFI of CD29 in CD226+ versus CD226- CD8+ T cells in CLL patients. **(G)** Cumulative data showing the MFI of CD29 in different subsets of CD226+ and CD226-CD8+ T cells in CLL patients. **(H)** Cumulative data of the frequency of CD160, TIGIT, PD-1, CD244, KLRG, EOMES, and GzmK expressing cells among CD226+ versus CD226 − $CD8^{+}CCR7^{-}$ T cells. **(I)** Cumulative data for T-bet, **(J)** FOXP3, **(K)** and RORγ t-expressing cells among CD226+ versus CD226-CD8 T cells in CLL patients. **(L)** Scatter plot of the correlation between the percentages of CD226+ CD8+ T cells and CD160+CD8+ T cells in CLL patients. (**M**) Representative flow plots, and **(N)** cumulative data of the frequency of CCR7 expressing cells among CD226+ versus CD226- CD8+ T cells. Cumulative data are presented as medians and interquartile ranges. The p-values were measured using linear regression (L), the Mann Whitney U test (D, F, I, J, K, N) or Kruskal–Wallis test followed by Dunn's post-hoc tests (B, C, E, G, H).

S Figure 2: (A) Cumulative data of the migratory capacity of CD226- versus CD226+CD8+ T cells in response to FBS. (**B**) Cumulative data of IL-8 and MIP-3α concentration in the plasma of HC and CLL patients. **(C)** PCA of the Euclidean distances derived from the cytokine and

chemokine profiles of plasma samples obtained from CLL patients, distinguishing between CLL patients with mutated and unmutated IgHV mutational statuses. **(D)** Cumulative MFI data of CD226+ $CD8^+$ T cells following stimulation with anti-CD3/CD28 in the presence or absence of recombinant human MIP-1α. (**E**) Cumulative data of soluble CD226 concentrations in the plasma of patients with CLL with low (0), intermediate (I/II), and high (III/IV) Rai stages. The p values were measured using the Mann Whitney U test (A,B), Wilcoxon test (D), and Kruskal–Wallis test followed by Dunn's post-hoc tests (E).

S Figure 3: Representing upregulated and downregulated transcriptional factors (TFs) in CD226+ versus their negative counterparts using decoupleR.
