# Supplementary material for: Polyfunctional CD8+ CD226+ RUNX2hi effector T cells are diminished in advanced stages of chronic lymphocytic leukemia
Source: Mol Oncol. 2025 Jan 7;19(5):1347–70. doi: 10.1002/1878-0261.13793 (PMC12077284; doi:10.1002/1878-0261.13793)
Supplement: Supplementary file 2 — Table S1. Clinical and demographic information of our study cohorts. [file MOL2-19-1347-s002.pdf]

Supplementary Table 1. Clinical and demographic information of our cohorts.

Age	Past Treatment for CLL if any	Zap 70	CD 38*	IGHV	Fish 11q22_3	Fish 12	Fish 13q14_3	Fish 13q14_2	Fish 17p	Rai stage
**60s**	FCR February 2018	ND	ND	ND	Normal	Trisomy	Normal	Normal	Normal	IV
**70s**		ND	ND	Mutated	Normal	Trisomy	Normal	Normal	Normal	I
**50s**		ND	ND	Unmutated	Normal	Trisomy	Normal	Normal	Normal	I
**50s**		ND	ND	ND	ND	ND	ND	ND	ND	I
**70s**	Rituximab + bendamustine 2017	ND	ND	ND	Normal	Normal	Abnormal	Normal	Normal	I
**70s**		ND	ND	ND	ND	ND	ND	ND	ND	I
**60s**		ND	ND	ND	Normal	Normal	Normal	Normal	Normal	III
**40s**		ND	ND	ND	ND	ND	ND	ND	ND	0
**70s**	Rituximab +bendamustine	ND	ND	ND	ND	ND	ND	ND	ND	0
**70s**	FCR 2013	ND	ND	ND	Normal	Normal	Abnormal	Normal	Normal	II
**80s**		ND	ND	Unmutated	Normal	Normal	Normal	Normal	Normal	III
**60s**		ND	ND	Unmutated	Normal	Normal	Normal	Normal	Normal	IV
**70s**		ND	ND	ND	ND	ND	ND	ND	ND	I
**30s**		ND	ND	Unmutated	Normal	Normal	Normal	Normal	Normal	II
**40s**		ND	ND	Mutated	ND	ND	ND	ND	ND	II
**70s**		ND	ND	Unmutated	Normal	Trisomy	Normal	Normal	Normal	I
**60s**		ND	ND	Mutated	ND	ND	ND	ND	Normal	II
**70s**		ND	ND	ND	ND	ND	ND	ND	ND	0
**80s**		ND	ND	ND	ND	ND	ND	ND	ND	0
**70s**		ND	ND	ND	ND	ND	ND	ND	ND	I
**60s**		Negative	ND	ND	ND	ND	ND	ND	ND	0
**50s**		ND	ND	Mutated	ND	ND	ND	ND	ND	0
**50s**		ND	ND	Mutated	ND	ND	ND	ND	ND	0
**50s**		ND	ND	Unmutated	Normal	Normal	Abnormal	Normal	Normal	IV
**70s**		ND	ND	ND	ND	ND	ND	ND	ND	0
**60s**		ND	ND	Indeterminant	ND	ND	ND	ND	ND	IV
**40s**		ND	ND	Mutated	Normal	Normal	Abnormal	Normal	Normal	I
**60s**		Positive	ND	ND	Normal	Normal	Normal	Normal	Abnormal	I
**70s**		Negative	ND	Mutated	Normal	Normal	Abnormal	Normal	Normal	I
**60s**		ND	ND	Mutated	ND	ND	ND	ND	ND	II

Supplementary Table 1. Clinical and demographic information of our cohorts.

**80s**		ND	ND	Unmutated	Abnormal	Normal	Normal	Normal	Normal	III
**60s**		ND	ND	Unmutated	ND	ND	ND	ND	ND	III
**60s**		ND	ND	ND	ND	ND	ND	ND	ND	0
**70s**	Chlorambucil July 2014 Ibrutinib October 2019	Positive	ND	Unmutated	Abnormal	Normal	Normal	Normal	Normal	IV
**70s**		Negative	ND	Unmutated	Abnormal	Normal	Abnormal	Abnormal	Normal	IV
**60s**	FCR 2012 Allogeneic stem cell transplant (with active chronic GVHD)	ND	ND	ND	Normal	Normal	Abnormal	Normal	Abnormal	IV
**80s**		ND	ND	Unmutated	Normal	Normal	Abnormal	Normal	Normal	III
**50s**		Positive	ND	ND	ND	ND	ND	ND	ND	0
**80s**		ND	ND	ND	ND	ND	ND	ND	ND	I
**80s**	Bendamustine+rituximab July 2016.	ND	ND	ND	Normal	Normal	Abnormal	Normal	Normal	IV
**60s**	FCR 2011	ND	ND	ND	Normal	Normal	Normal	Normal	Abnormal	II
**70s**	Chlorambucil/obinituzumab	ND	ND	ND	Normal	Trisomy	Normal	Normal	Normal	I
**70s**		Positive	ND	ND	ND	ND	ND	ND	ND	0
**30s**		ND	ND	Unmutated	Abnormal	Normal	Abnormal	Normal	Normal	II
**50s**		ND	ND	ND	Normal	Normal	Normal	Normal	Normal	I
**70s**		ND	ND	Unmutated	Normal	trisomy	Normal	Normal	Normal	II
**80s**		ND	ND	ND	ND	ND	ND	ND	ND	I
**70s**		ND	ND	Mutated	Normal	Normal	Abnormal	Abnormal	Normal	0
**60s**		Positive	ND	ND	ND	ND	ND	ND	Abnormal	I
**70s**		ND	ND	ND	ND	ND	ND	ND	Normal	0
**60s**		ND	ND	ND	Normal	Normal	Abnormal	Normal	Normal	II

Supplementary Table 1. Clinical and demographic information of our cohorts.

**60s**		ND	ND	ND	Normal	Trisomy	Abnormal	Normal	Normal	III
**70s**		Positive	ND	ND	ND	ND	ND	ND	ND	0
**50s**	FCR 2018	Positive	ND	Unmutated	Normal	Normal	Normal	Abnormal	Normal	I
**60s**		Negative	ND	ND	ND	ND	ND	ND	ND	0
**60s**		ND	ND	Unmutated	Normal	Trisomy	Normal	Normal	Normal	II
**70s**		ND	ND	ND	ND	ND	ND	ND	ND	0
**70s**		ND	ND	ND	ND	ND	ND	ND	ND	0
**60s**		ND	ND	Unmutated	Normal	Normal	Abnormal	Normal	Normal	III
**70s**		ND	ND	ND	ND	ND	ND	ND	ND	0
**60s**		ND	ND	ND	ND	ND	ND	ND	ND	I
**60s**		ND	ND	ND	ND	ND	ND	ND	ND	I
**70s**		ND	ND	Mutated	ND	ND	ND	ND	Abnormal	III
	Healthy	controls								
**40s**	HC-01	N/A	N/A	N/A	N/A	N/A	N/A	N/A	N/A	N/A
**70s**	HC-02	N/A	N/A	N/A	N/A	N/A	N/A	N/A	N/A	N/A
**60s**	HC-03	N/A	N/A	N/A	N/A	N/A	N/A	N/A	N/A	N/A
**70s**	HC-04	N/A	N/A	N/A	N/A	N/A	N/A	N/A	N/A	N/A
**50s**	HC-05	N/A	N/A	N/A	N/A	N/A	N/A	N/A	N/A	N/A
**60s**	HC-06	N/A	N/A	N/A	N/A	N/A	N/A	N/A	N/A	N/A
**70s**	HC-07	N/A	N/A	N/A	N/A	N/A	N/A	N/A	N/A	N/A
**50s**	HC-08	N/A	N/A	N/A	N/A	N/A	N/A	N/A	N/A	N/A
**70s**	HC-09	N/A	N/A	N/A	N/A	N/A	N/A	N/A	N/A	N/A
**40s**	HC-10	N/A	N/A	N/A	N/A	N/A	N/A	N/A	N/A	N/A
**50s**	HC-11	N/A	N/A	N/A	N/A	N/A	N/A	N/A	N/A	N/A
**60s**	HC-12	N/A	N/A	N/A	N/A	N/A	N/A	N/A	N/A	N/A
**60s**	HC-13	N/A	N/A	N/A	N/A	N/A	N/A	N/A	N/A	N/A
**60s**	HC-14	N/A	N/A	N/A	N/A	N/A	N/A	N/A	N/A	N/A
**60s**	HC-15	N/A	N/A	N/A	N/A	N/A	N/A	N/A	N/A	N/A
**70s**	HC-16	N/A	N/A	N/A	N/A	N/A	N/A	N/A	N/A	N/A
**60s**	HC-17	N/A	N/A	N/A	N/A	N/A	N/A	N/A	N/A	N/A
**80s**	HC-18	N/A	N/A	N/A	N/A	N/A	N/A	N/A	N/A	N/A
**60s**	HC-19	N/A	N/A	N/A	N/A	N/A	N/A	N/A	N/A	N/A
**70s**	HC-20	N/A	N/A	N/A	N/A	N/A	N/A	N/A	N/A	N/A
**60s**	HC-21	N/A	N/A	N/A	N/A	N/A	N/A	N/A	N/A	N/A
**70s**	HC-22	N/A	N/A	N/A	N/A	N/A	N/A	N/A	N/A	N/A
**60s**	HC-23	N/A	N/A	N/A	N/A	N/A	N/A	N/A	N/A	N/A
**50s**	HC-24	N/A	N/A	N/A	N/A	N/A	N/A	N/A	N/A	N/A
**70s**	HC-25	N/A	N/A	N/A	N/A	N/A	N/A	N/A	N/A	N/A

Supplementary Table 1. Clinical and demographic information of our cohorts.

Treatment: (FCR: Fludarabine, cyclophosphamide and rituximab)
Not determined (ND)
Healthy control (HC)
Not applicable (N/A)
